# Base-Free Catalytic Hydrogen Production from Formic Acid Mediated by a Cubane-Type Mo_3_S_4_ Cluster Hydride

**DOI:** 10.1021/acs.inorgchem.2c02540

**Published:** 2022-10-14

**Authors:** Eva Guillamón, Iván Sorribes, Vicent S. Safont, Andrés G. Algarra, M. Jesús Fernández-Trujillo, Elena Pedrajas, Rosa Llusar, Manuel G. Basallote

**Affiliations:** † Departament de Química Física i Analítica, Universitat Jaume I, Av. Sos Baynat s/n, 12071Castelló, Spain; ‡ Departamento de Ciencia de los Materiales e Ingeniería Metalúrgica y Química Inorgánica, Instituto de Biomoléculas (INBIO), Facultad de Ciencias, Universidad de Cádiz, Apartado 40, Puerto Real, 11510Cádiz, Spain

## Abstract

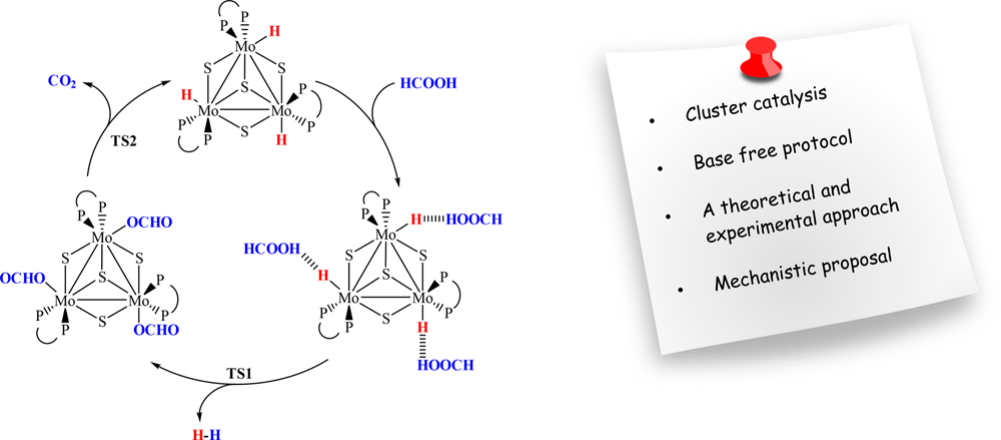

Formic acid (FA) dehydrogenation is an attractive process in the implementation of a hydrogen economy. To make this process greener and less costly, the interest nowadays is moving toward non-noble metal catalysts and additive-free protocols. Efficient protocols using earth abundant first row transition metals, mostly iron, have been developed, but other metals, such as molybdenum, remain practically unexplored. Herein, we present the transformation of FA to form H_2_ and CO_2_ through a cluster catalysis mechanism mediated by a cuboidal [Mo_3_S_4_H_3_(dmpe)_3_]^+^ hydride cluster in the absence of base or any other additive. Our catalyst has proved to be more active and selective than the other molybdenum compounds reported to date for this purpose. Kinetic studies, reaction monitoring, and isolation of the [Mo_3_S_4_(OCHO)_3_(dmpe)_3_]^+^ formate reaction intermediate, in combination with DFT calculations, have allowed us to formulate an unambiguous mechanism of FA dehydrogenation. Kinetic studies indicate that the reaction at temperatures up to 60 °C ends at the triformate complex and occurs in a single kinetic step, which can be interpreted in terms of statistical kinetics at the three metal centers. The process starts with the formation of a dihydrogen-bonded species with Mo–H···HOOCH bonds, detected by NMR techniques, followed by hydrogen release and formate coordination. Whereas this process is favored at temperatures up to 60 °C, the subsequent β-hydride elimination that allows for the CO_2_ release and closes the catalytic cycle is only completed at higher temperatures. The cycle also operates starting from the [Mo_3_S_4_(OCHO)_3_(dmpe)_3_]^+^ formate intermediate, again with preservation of the cluster integrity, which adds our proposal to the list of the infrequent cluster catalysis reaction mechanisms.

## Abstract 2

Molybdenum complexes are slowly emerging as an alternative to noble metals for the catalytic dehydrogenation of FA. The present protocol operates through a cluster catalysis mechanism in the absence of additives.

## Introduction

Hydrogen storage methods are rapidly emerging to provide answers to the intermittent energy supply inherent to renewable sources. Chemical hydrogen carriers in which hydrogen is covalently bound and can be catalytically released have been proposed as an interesting alternative.^1^ Nowadays, formic acid (FA) is an attractive storing hydrogen system, and the possibility of a direct reversible hydrogenation of CO_2_ to FA and *vice versa* represents a vector for “green” hydrogen storage. Although the first report on FA dehydrogenation appeared in the late 1960s, the FA potential as a liquid hydrogen carrier was first highlighted in 2008 by Beller et al. and Laurenczy et al.^2−4^ Since then, a plethora of homogeneous mononuclear catalysts have emerged based on ruthenium, iridium, and rhodium well-defined complexes or in situ generated species in the presence of phosphine, aminophosphine, diimine, carbene, and N-donor heterocyclic ligands.^5,6^ The structure of the ligand has a strong influence on the catalytic activity.^7^ Top performances have been reported for a Ru(I) hydrido complex bearing a 9H-acridine pincer PNP ligand at 65–95 °C in neat FA and for half-sandwich pyridyl-imidazolyl Ir(II) complexes at 70 °C in water.^8,9^ Bis-N-heterocyclic carbene NHC Rh(III) complexes are also effective catalysts for the selective FA dehydrogenation in aqueous solutions at 100 °C.^11^ Exceptionally, these three complexes reach remarkable activities in the absence of additives, unlike most transition metal homogeneous catalysts. The design of green catalysts based on abundant metals operating under additive free conditions remains a challenge nowadays.

In the past decade, several first row transition metal catalysts have emerged to overcome the limitations and price of noble metals.^12^ Up to date, the best results reported for the catalytic dehydrogenation of FA using an earth-abundant transition metal based catalyst have been obtained by Schneider et al. using a mononuclear Fe(II) hydrido complex functionalized with pincer PN^H^P, carbonyl, and formate ligands.^13^ This iron catalyst affords TOF values in the 1400–200 000 h^–1^ range at 80 °C in dioxane with the activity depending on the nature of the Lewis acid employed as a cocatalyst. The utility of iron complexes as catalysts for the generation of hydrogen from FA was first established by the group of Beller.^14^ Milstein and co-workers reported TOFs of 500 h^–1^ for pincer-supported PNP iron catalysts in the presence of NEt_3_ in THF at 40 °C.^15^ Previously, Beller and Laurenczy had described an effective “in situ” generated Fe(II) catalyst in the presence of a tetradentate phosphine that releases hydrogen from FA in propylene carbonate with no further additives or base and with a TOF of 5800 h^–1^ at 80 °C.^16^ More recently, promising results were also obtained with well-defined pincer-type PNP cobalt and manganese complexes, although TOFs were inferior to those achieved with iron and basic additives.^17,18^ In 2020, Beller et al. showed that *N*,*N*′-imidazoline-based manganese complexes were also effective FA dehydrogenation catalysts in water:triglyme at 92.5 °C under KOH basic conditions, with TOFs ranging between 6 and 193 h^–1^.^19^ Systems based on other first row transition metals, i.e., Ni and Cu, are limited to a few examples, and their performance, under basic conditions, is inferior to that of their lighter counterparts.^20,21^ With very few exceptions, first row transition metal catalysts require additives to reach good activities in FA dehydrogenation.

**Scheme 1:**
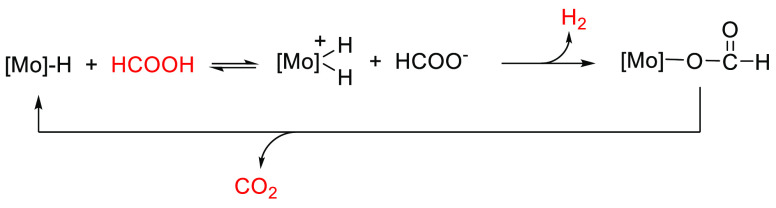
Simplified Mechanism for the FA Dehydrogenation Catalyzed by Cp*Mo(PMe_3_)_2_(CO)H.^23^

The use of second row transition metals other than noble metals is almost unexplored. In 2002, the cyclopentadienyl molybdenum hydride compound Cp*Mo(PMe_3_)_2_(CO)H was presented for FA dehydrogenation by Parkin and co-workers.^22^ More than a decade later, this group has reinvestigated the catalytic activity of this complex and also extended its study to other members of the Cp^R^Mo(PMe_3_)_3–*x*_(CO)_*x*_H (Cp^R^ = Cp, Cp*; *x* = 0, 1, 2, 3) series.^23^ Hydrogen evolution from FA using Cp^R^Mo(PMe_3_)_2_(CO)H proceeds with a TOF of 54 h^–1^ at 100 °C in benzene without a base. The essential features of the mechanism are shown in Scheme 1. Remarkably, even though CO_2_ and H_2_ are the main products of this reaction, methanol and methyl formate are also observed. Production of methanol occurs through FA disproportionation, while subsequent esterification affords the methyl formate. More recently, Alberico, Beller and co-workers have reported a series of molybdenum complexes containing aliphatic PN^H^P pincer ligands which also catalyze both FA disproportionation and dehydrogenation.^24^ To the best of our knowledge, the above molybdenum complexes provide the only reported examples of catalytically active molybdenum compounds for the liberation of hydrogen from FA.

**Figure 1:**
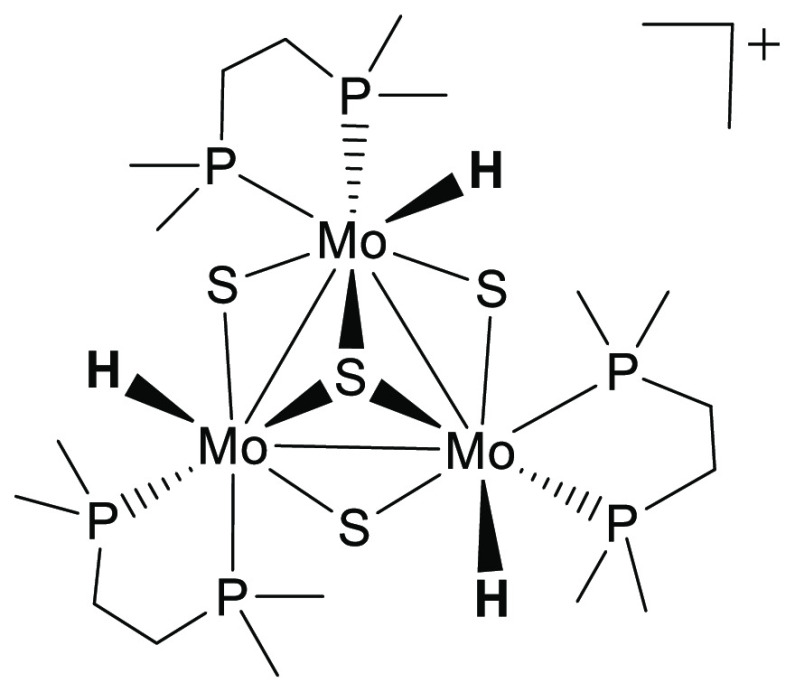
Structure of the [Mo_3_S_4_H_3_(dmpe)_3_]^+^ cluster cation.

In 2012, some of us, in collaboration with Beller’s group, reported that the cubane-type [Mo_3_S_4_H_3_(dmpe)_3_]^+^ (dmpe = 1,2-(bis)dimethyl-phosphinoethane) cluster hydride, represented in Figure 1, catalyzes the transfer hydrogenation of nitroarenes to anilines using an azeotropic mixture of FA and Et_3_N in THF at 70 °C with full conversion and high selectivity.^25^ Previous kinetic and theoretical studies by some of us on the reactivity of these molybdenum and tungsten [M_3_S_4_H_3_(diphosphine)_3_]^+^ (M = Mo, W) hydrides toward acids led us to postulate the formation of dihydrogen species prior to hydride substitution.^26−28^

In a recent in-depth theoretical investigation on the transfer hydrogenation mechanism of nitroarenes, we confirmed the presence of adducts with Mo–H···HOOCH interactions from which hydrogen is transferred to the organic substrates, which results in the formation of a formate-substituted cluster that regenerates the initial cluster hydride through a β-hydride elimination accompanied by CO_2_ release.^29^ Our mechanistic proposal shares common basic features with that of Parkin on FA dehydrogenation by well-defined Mo(II) hydrides, shown in Scheme 1, except for the nature of the Mo(H_2_) species which Parkin identified as a dihydride complex, while we postulate the formation of Mo–H···HOOCH species. Motivated by these resemblances, we decided to investigate the potential of the [Mo_3_S_4_H_3_(dmpe)_3_]^+^ cluster cation as a catalyst for the dehydrogenation of FA.

The ideal scenario for hydrogen generation from FA contemplates a selective process catalyzed by earth abundant metals in the absence of other additives. Herein, we present a cluster catalysis mechanism that fulfils those criteria. The cubane-type [Mo_3_S_4_H_3_(dmpe)_3_](BPh_4_) hydride salt liberates hydrogen free of carbon monoxide from FA in propylene carbonate with no additives. Although activities are only moderate, this contribution illustrates for the first time the potential of molybdenum clusters as catalysts for hydrogen evolution from a chemical carrier such as FA. A mechanistic proposal based on kinetic experiments and reaction monitoring combined with DFT calculations is presented, laying the foundations for further improvements.

## Results and Discussion

### Catalytic Performance

Cluster catalysis mechanisms in FA dehydrogenation represent an unexplored field. In contrast, trinuclear Ru_3_(CO)_9_ carbonyl clusters efficiently catalyze the reverse reaction, being the only example of cluster catalysis in this area; that is, the cluster unit is preserved during the process.^30^ The potential of cubane-type molybdenum clusters as catalysts for the transfer hydrogenation of organic substrates has led us to investigate the generation of hydrogen from FA. Initially, the reaction was tested at different initial acid concentrations and temperatures. Gas evolution was observed in propylene carbonate for temperatures higher than 100 °C. A crucial dependence of the activity on the initial acid concentration was observed. The results are summarized in Table 1.

**Table 1:**
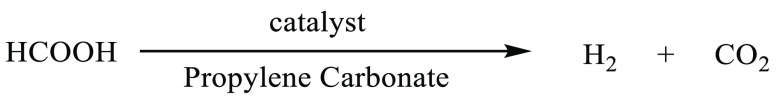
Catalytic Dehydrogenation of Formic Acid under Different Reaction Conditions^a^

entry	HCOOH (mmol)	temperature (°C)	gas volume (mL)^b^	time (h)	conversion (%)	TON	TOF (h^–1^)
1	10	130	7.8	3.5	1	21	6
2	2	130	82.2	7	84	222	32
3	2	100	9.8	8	10	27	3
4	2	110	22.4	9	23	61	7
5	2	120	79.2	8	81	214	27
6	1	120	42.8	1	87	116	116

a 1.5 mL of propylene carbonate and 8.64 μmol of [Mo_3_S_4_H_3_(dmpe)_3_](BPh_4_) catalyst were used.

b H_2_ + CO_2_ volume monitored with manual burets and corrected by the blank volume (2.6 mL for entry 1; 0.4 mL for entries 2, 4–6; and 0 mL for entry 3).

A good performance was obtained at 120 °C using 1 mmol of FA with a TOF of 116 h^–1^ (Table 1, entry 6). Although TOF increases at higher temperatures (Table 1, entries 2–5), it should be noted that heating the system would favor the dehydration of FA to afford H_2_O and CO, which is detrimental for fuel cell applications. Noticeably, no CO (as a result of FA dehydration) was detected by GC beyond the expected 1:1 ratio of H_2_ and CO_2_ at 120 °C (Figure S1, SI). An interesting point is the decrease in the catalyst activity at a higher acid concentration (Table 1, entries 1 and 2), which we attribute to the loss of efficiency of the catalyst at lower pH. To overcome this limitation, FA was added directly to the system without recovering the catalyst, and the results are summarized in Table 2. The corresponding curves of the gas evolution vs time are provided as SI (Figure S2). The catalyst activity decreases after the second run (Table 2, entries 1 and 2); however, the protocol can be applied up to four times, although longer reaction times are needed (Table 2, entries 2–4). After this (Table 2, entry 5), the catalyst substantially reduces its activity.

**Table 2:**
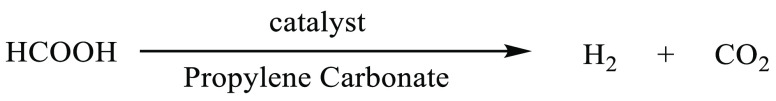
Catalyst Recycling Experiments^a^

run	gas volume^b^ (mL)	time^c^ (min)	conversion (%)	TON (1 h)
1	42.8	70	88	104
2^d^	44.0	80	90	105
3^d^	40.8	90	84	72
4^d^	38.4	150	79	34
5^d^	3.8	70	8	8

a Reaction conditions: HCOOH (1 mmol), catalyst (8.64 μmol), propylene carbonate (1.5 mL), *T* = 120 °C.

b Volume corrected by the blank volume (0.4 mL). Experiments were performed at least twice (standard deviation <10%).

c Time required to completeness.

d After each run, the reaction mixture was cooled to r.t. and then 1 mmol of HCOOH was added.

Next, the cluster integrity during the catalytic process was monitored by electrospray mass spectrometry (ESI-MS) at different reaction times (Figure S3, SI). In all cases, we observed that the Mo_3_S_4_ cluster unit remains intact as well as the coordinated diphosphines, while the outer hydride ligands are sequentially substituted by formate ligands. After 20 min and at the end of reaction, the predominant species are [Mo_3_S_4_H(OH)_2_(dmpe)_3_]^+^ (*m*/*z* = 898) and the trisubstituted [Mo_3_S_4_(OCHO)_3_(dmpe)_3_]^+^ (*m*/*z* = 1001) formate cluster complexes. Minor peaks corresponding to the partial substitution of the outer hydrido or hydroxo ligands by formate groups are also observed. Due to the presence of traces of water in the solvent, these substitution processes can also occur during the ESI-MS recording. Thus, the cluster unit is preserved, fulfilling the basis of the criteria of cluster catalysis.^31^ Nevertheless, the decrease in the TON values observed in successive additions and cluster monitoring by ESI-MS suggests the existence of some pathway for degradation of the catalyst toward lower nuclearity species that was not explored in detail.

### Kinetic and DFT Studies on the Mechanism of Formation of the Triformate Cluster

To obtain additional information about the mechanism of the catalytic process, kinetic studies on the reaction of the hydride cluster [Mo_3_S_4_H_3_(dmpe)_3_]^+^ with FA were carried out by recording the changes in the UV–vis spectrum using a conventional spectrophotometer. As pointed out in the previous section, no gas evolution is observed at temperatures lower than 100 °C, which means that the catalytic cycle is not completed at lower temperatures. However, preliminary experiments at 25 and 60 °C clearly showed that the hydride cluster reacts with an excess of FA, and the nature of the resulting product was established as [Mo_3_S_4_(OCHO)_3_(dmpe)_3_]^+^ on the basis of the NMR and ESI-MS spectra (Figures S8–S11, SI). The same reaction product was found to be formed in propylene carbonate and acetonitrile solutions. Thus, the reaction occurring under those conditions can be represented by eq 1, which is similar to those previously reported for the reaction of related hydride clusters with other acids.^26−28,32^ Although the reaction involves evolution of H_2_, it occurs under stoichiometric conditions, and therefore the amount of gas formed is too small to be detected with the experimental setup used for the catalytic experiments.
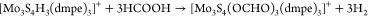
1

**Figure 2:**
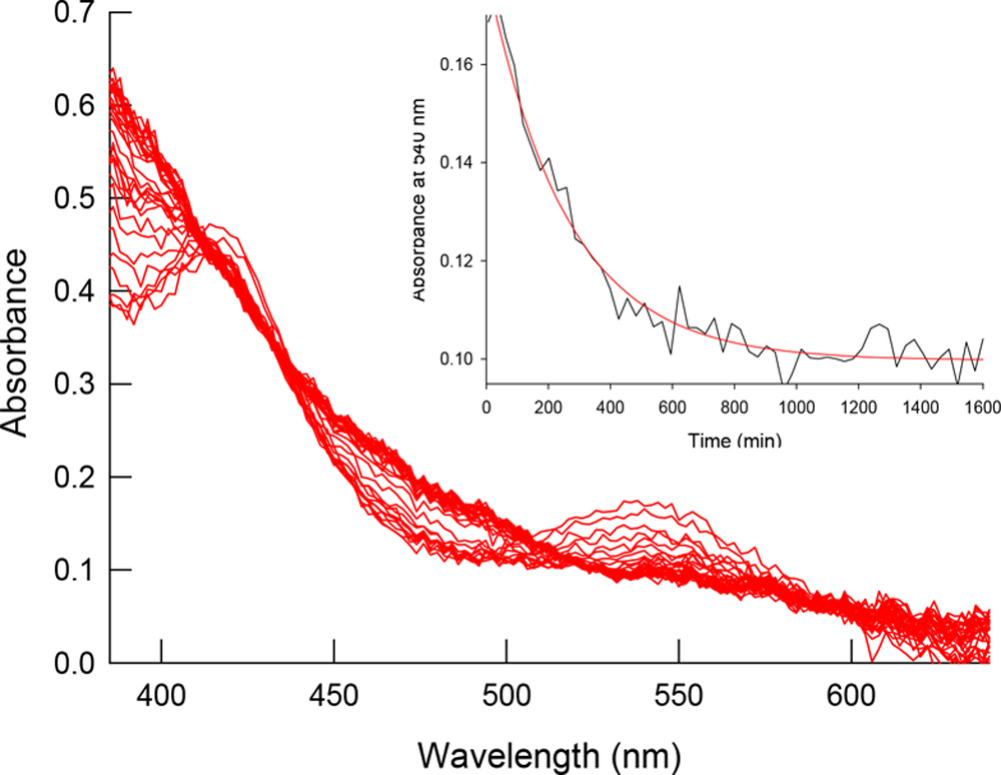
Spectral changes observed for the reaction of complex [Mo_3_S_4_H_3_(dmpe)_3_](BPh_4_) (1.5 × 10^–4^ M) with HCOOH (0.08 M) in acetonitrile solution at 25.0 °C. Inset: trace at 540 nm (black) showing the fit to a single kinetic step (red).

The kinetics of reaction of [Mo_3_S_4_H_3_(dmpe)_3_]^+^ with FA was then studied not only in propylene carbonate but also in acetonitrile solution to obtain results comparable with those previously reported for the reaction with other hydride clusters. The spectral changes are quite similar in both solvents (Figure 2 and Figure S5, SI) and clearly show the disappearance of the characteristic band of the cluster at 550 nm.

**Figure 3:**
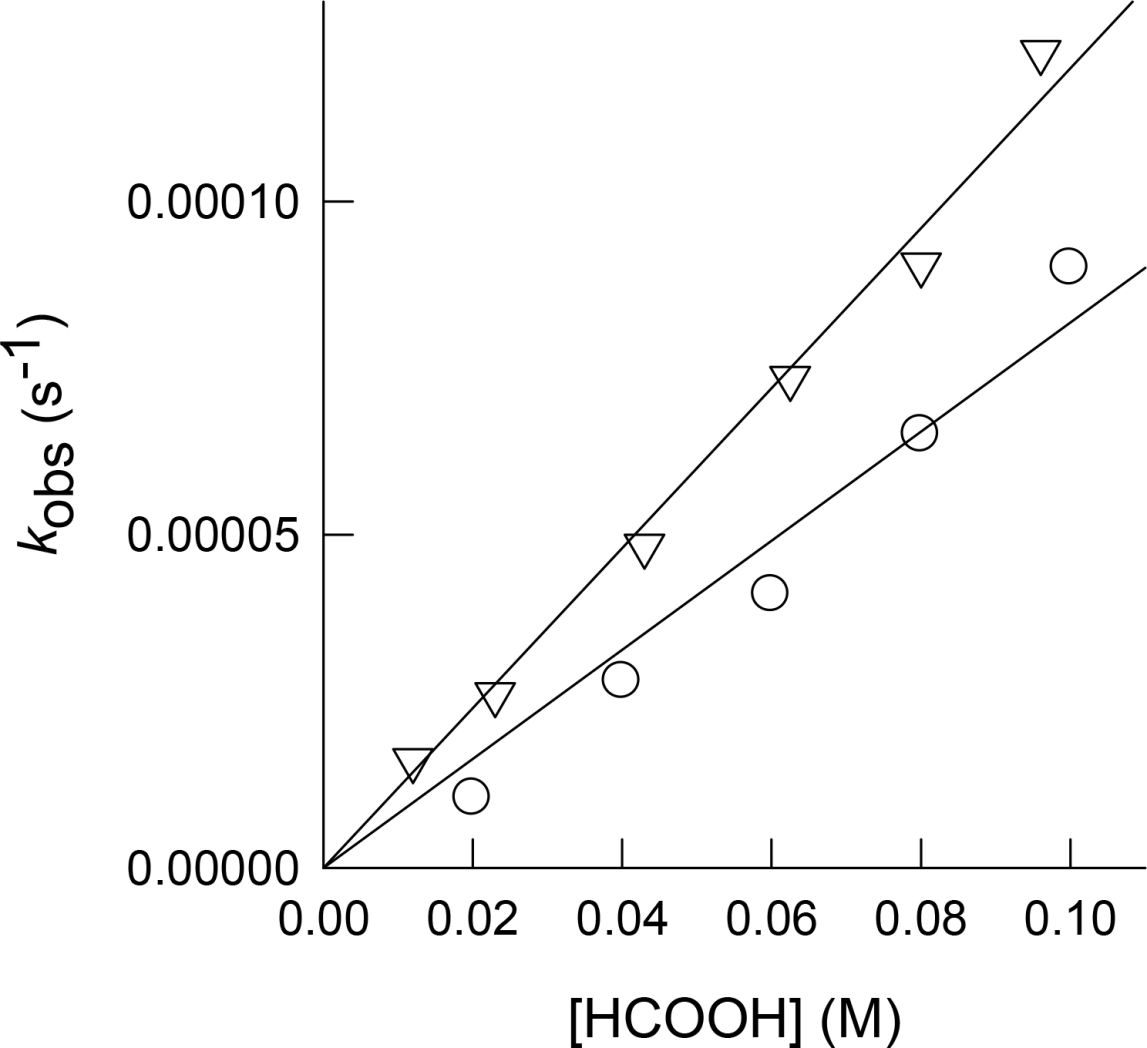
Plot of the rate constants dependence with the HCOOH concentration for the reaction of cluster [Mo_3_S_4_H_3_(dmpe)_3_]^+^ with HCOOH in acetonitrile (circles) and propylene carbonate (triangles) solutions at 25 °C.

The spectral changes could be fitted in all cases to a single kinetic step with values of the observed rate constant that change linearly with the FA concentration (Figure 3). The values derived for the second order rate constant in acetonitrile and propylene carbonate at 25 °C, (8.2 ± 0.5) × 10^–4^, and (12.0 ± 0.3) × 10^–4^ M^–1^ s^–1^, respectively, indicate that there are no large kinetic differences in both solvents. In the case of propylene carbonate, the kinetics were also studied at 60 °C to check the influence of temperature (Figure S6), SI and a modest acceleration was observed. Indeed, the rate constant value of (1.36 ± 0.04) × 10^–2^ M^–1^ s^–1^ is only 1 order of magnitude faster than at 25 °C.

As separate kinetic steps could be resolved for the reaction at the three metal centers in some previous studies of related hydride clusters with acids, we checked by NMR and ESI-MS the nature of the reaction product after two to three half-times and found that [Mo_3_S_4_(OCHO)_3_(dmpe)_3_]^+^ is the major species under those conditions, which means that the single kinetic step resolved for the reaction of [Mo_3_S_4_H_3_(dmpe)_3_]^+^ with FA corresponds to the reaction occurring at the three metal centers (eq 1) with statistically controlled kinetics, i.e., rate constants in a 3:2:1 ratio for the reactions at the three metal centers. The observation of a single kinetic step for sequential reactions at the three metal centers of this kind of cluster is well illustrated in the literature.^33^ The formation of H_2_ in the reaction was confirmed by the observation of a signal at 4.55 ppm in the NMR spectra recorded after the addition of FA (Figure S4, SI).

The present kinetic results for the reaction of [Mo_3_S_4_H_3_(dmpe)_3_]^+^ with FA can then be interpreted in terms of the simplified mechanism in eqs 2 and 3, where the initial step is a fast pre-equilibrium of formation of an adduct with a Mo–H···HOOCH interaction, which is followed by the rate-determining direct formation of a formate product. The same mechanism would be repeated at the three metal centers with statistical kinetics. This mechanism is similar to that previously proposed for the reaction of other hydride complexes with different acids, including both mono- and trinuclear Mo complexes.^28,34,35^ The rate law for this mechanism is given by eq 4, which simplifies to the experimental rate law with *k* = *k*_H_2__ × *K*_dhb_ when 1 ≫ *K*_dhb_ [HCOOH].
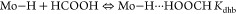
2
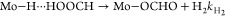
3
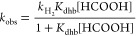
4

**Figure 4:**
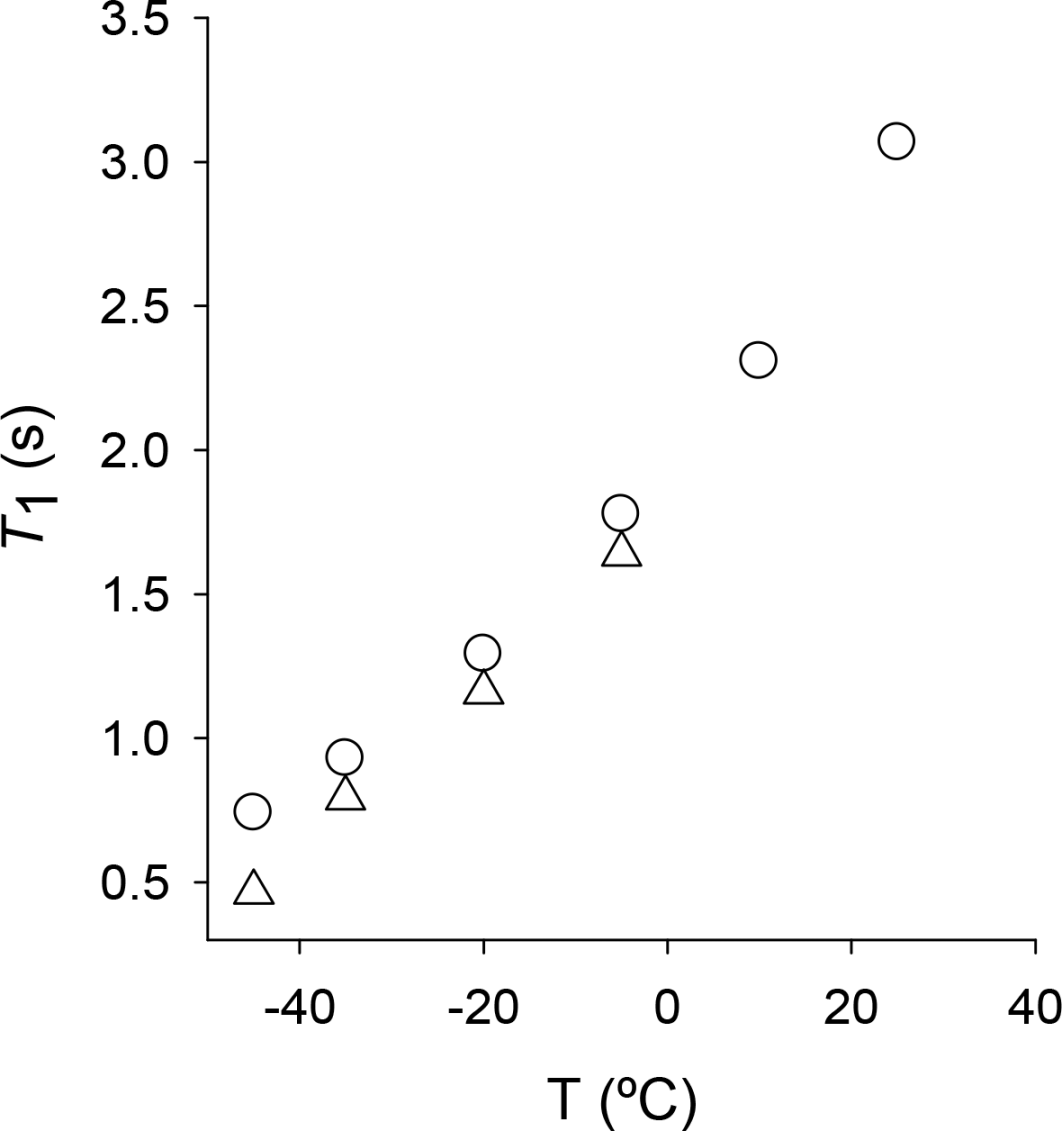
Temperature dependence of the *T*_1_ value for the hydride signal of complex [Mo_3_S_4_H_3_(dmpe)_3_]^+^ in propylene carbonate solution. The circles correspond to the data for the complex alone and the triangles to the complex with an excess of HCOOH (25 equiv).

According to this mechanism, the reaction of [Mo_3_S_4_H_3_(dmpe)_3_]^+^ with FA starts with the interaction of the hydrido [Mo_3_S_4_H_3_(dmpe)_3_]^+^ cluster with the acid to form a Mo–H···HOOCH dihydrogen-bonded species (eq 2). In agreement with these expectations, the *T*_1_ values of the proton NMR hydride signal decrease in the presence of acid in both solvents (Figure 4 and Figure S7, SI), which provides strong evidence for the initial attack by the acid at the hydride ligands. Although the minimum *T*_1_ values cannot be reached at temperatures higher than the freezing point of the solvents, the data clearly show that acid addition decreases the relaxation time as a consequence of the proximity of the hydride and the proton in the Mo–H···HOOCH species. Decreases of *T*_1_ values of the same order of magnitude have been previously found for the interaction of related clusters with acids.^27^ The important role of these dihydrogen bonded species is further substantiated by the fact that the related [Mo_3_S_4_Cl_3_(dmpe)_3_](BPh_4_) cluster, which is unable to form dihydrogen bonds with FA, only shows very low activity as a catalyst with a TON of 1.25 after 6 h.

**Scheme 2:**
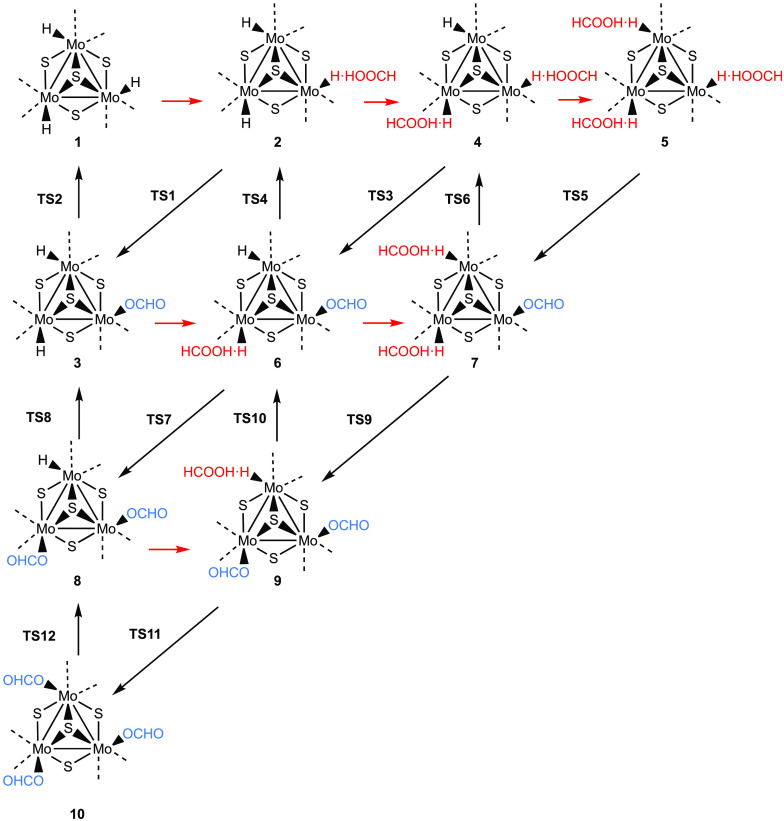
Different Molybdenum Cluster Species Involved in the Formic Acid Decomposition through a Multiple Metal Mechanism

The proposed mechanism for the dehydrogenation reaction in eq 1 is further supported by DFT calculations. In Scheme 2, we present the different molybdenum cluster species that can participate in the formation of [Mo_3_S_4_(OCHO)_3_(dmpe)_3_]^+^. In this scheme, red arrows correspond to the formation of MoH···HOOCH species, due to formic acid interaction with the cluster; black arrows correspond either to dehydrogenation processes (slanted arrows with odd-numbered transition states) or to decarboxylation steps (vertical arrows with even-numbered transition states). The carboxylate ligands (OCHO) formed after dehydrogenation of the dihydrogen species are shown in blue. The total Gibbs free energies of the calculated stationary points are reported in Table S2 (SI).

**Figure 5:**
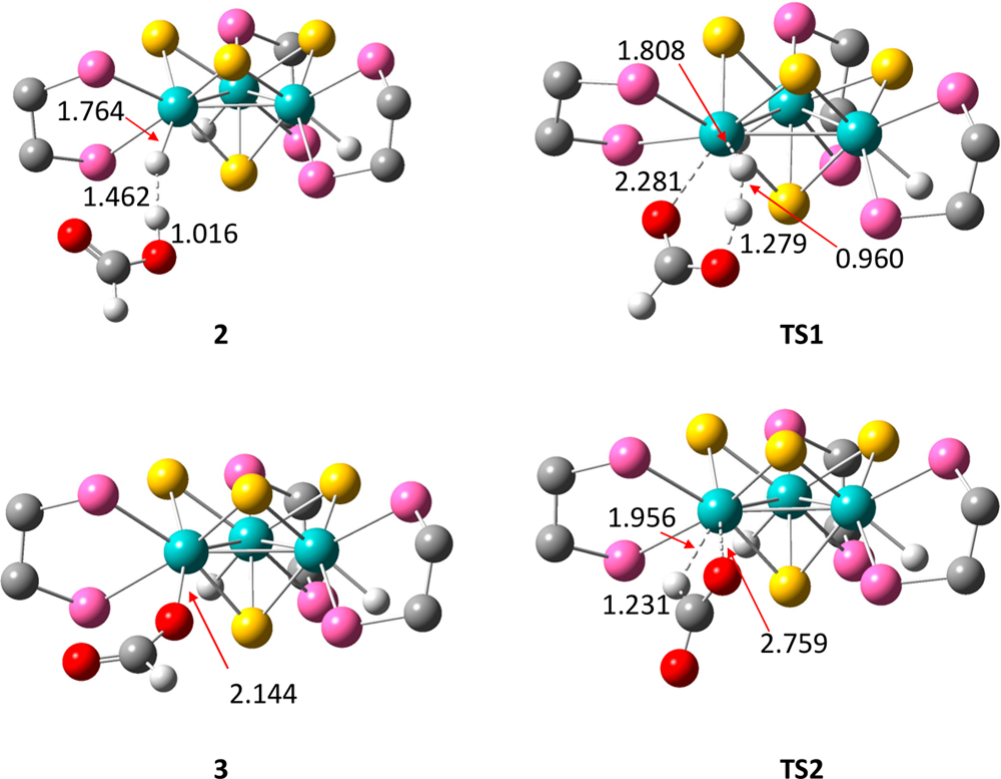
DFT-optimized structures of **2**, **3**, **TS1**, and **TS2**. For clarity, only the skeleton of the dmpe ligands was drawn. Distances are given in Å. Color code: Mo (Cyan), S (yellow), P (pink), O (red), C (gray), H (white).

The DFT-optimized structures associated with the first H_2_-release step of the process, i.e., the conversion of **2** into **3** + H_2_ via **TS1**, are included in Figure 5. The H···H distance of 1.462 Å in the MoH···HOOCH fragment of **2** decreases down to 0.960 Å in the **TS1** transition state. This interaction is accompanied by a Mo–H bond distance elongation of 0.019 Å on going from **1** (*d*_Mo–H_ = 1.745 Å) to **2** and of 0.044 Å on going from **2** to **TS1**. Similar tendencies in the H···H and M–H distances are found during the interaction of other hydrido Mo_3_S_4_ clusters with acids, i.e., HCl, with minor differences associated with the lower acidity of FA with respect to HCl. Mononuclear [Cp*Mo(dppe)H_3_] polihydrido clusters also interact with acids such as trifluoroethanol (HOR^F^) to form MoH···HOR^F^ species with the shortest optimized H···H distances ranging between 1.65 and 1.94 Å, that is, slightly longer that the analogous distance of 1.462 Å optimized for **2**.^35^ The stabilization energy due to the formation of MoH···HOOCH interactions in **2** at 25 °C of −0.60 kcal/mol compares with the values calculated for the above-reported molybdenum hydrides. In all cases, the stability of the adduct decreases upon increasing the temperature due to the unfavorable entropy contribution. This is in agreement with the experimental T1 values represented in Figure 4.

**Figure 6:**
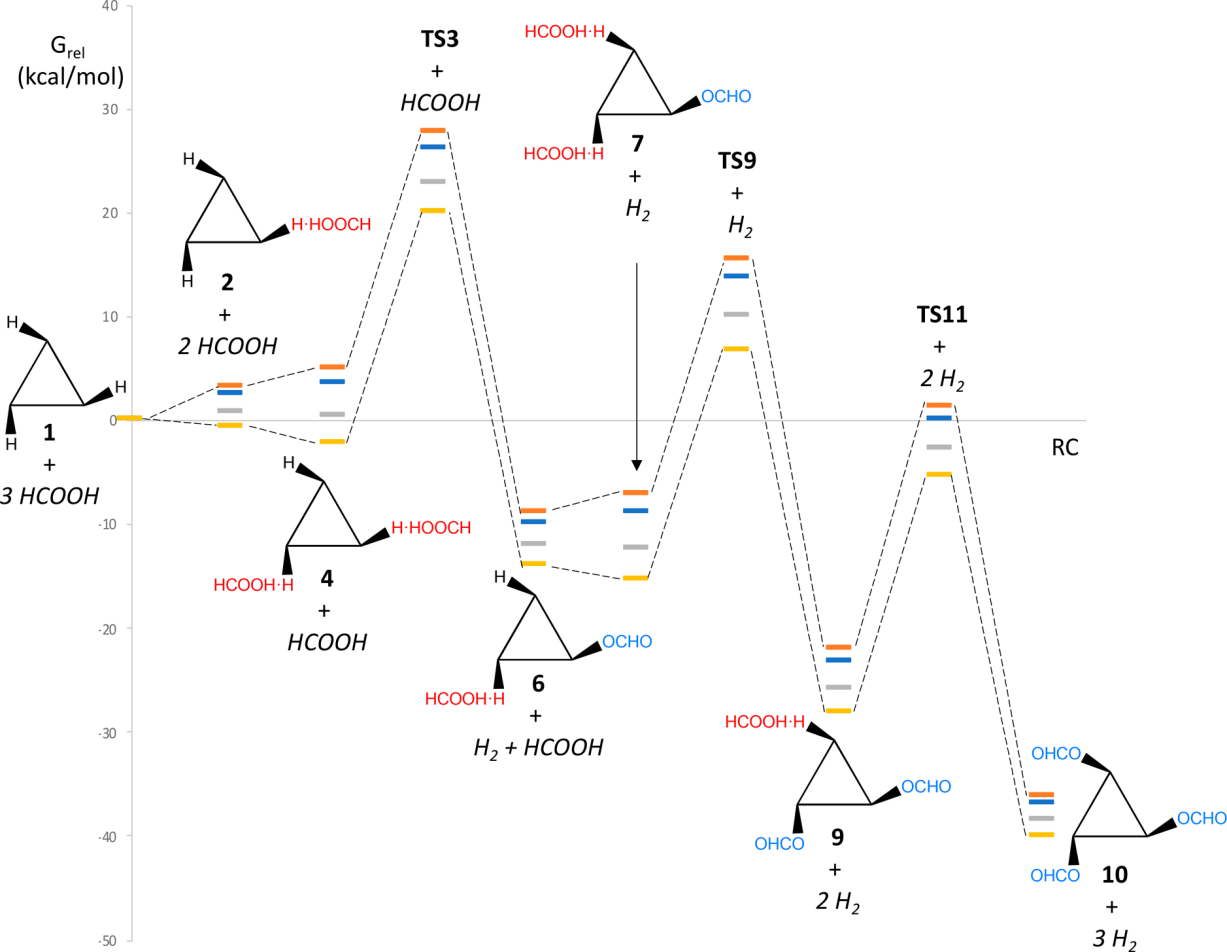
Free energy profile for the reaction of [Mo_3_S_4_H_3_(dmpe)_3_]^+^ (**1**) with FA to form [Mo_3_S_4_(OCHO)_3_(dmpe)_3_]^+^ in propylene carbonate solution at different temperatures. Color code: 25 °C (yellow), 60 °C (gray), 100 °C (blue), and 120 °C (orange).

The energy profile in Figure 6 shows the conversion of [Mo_3_S_4_H_3_(dmpe)_3_]^+^ (**1**) into [Mo_3_S_4_(OCHO)_3_(dmpe)_3_]^+^ (**10**) along the pathway with the lowest barrier: the one going through intermediates **2, 4**, 6, **7**, and **9** (see Table S3 for the *G*_rel_ values). For simplicity, other possible pathways in Scheme 2 have not been included here but can be found in the SI. Given the large difference of temperature between the kinetic and the catalytic experiments, the figure shows the free energy values at different temperatures between 25 and 120 °C. The overall process is thermodynamically favored at all temperatures and occurs with close activation energies for the three consecutive steps, in agreement with the experimental observation of statistical kinetics. The pathway shown in Figure 6 has barriers of 22.3, 22.2, and 22.8 kcal mol^–1^ at 25 °C for the reactions at the three metals, and of 22.4, 22.4, and 23.0 kcal mol^–1^ at 60 °C. Moreover, the activation barrier derived from the measured rate constants (Δ*G*^‡^ = 21.4 kcal mol^–1^ at 25 °C and 22.4 kcal mol^–1^ at 60 °C) agree well, within experimental and computational errors, with the computed barrier for the reaction at the third metal center (Δ*G*^‡^ = 22.8 kcal mol^–1^ at 25 °C and 23.0 kcal mol^–1^ at 60 °C), which is the one corresponding to the observed rate constant when the simplification caused by the statistical kinetics operates.^33^

### Closing the Catalytic Cycle: Elimination of CO_2_ from [Mo_3_S_4_(OCHO)_3_(dmpe)_3_]^+^

**Scheme 3:**
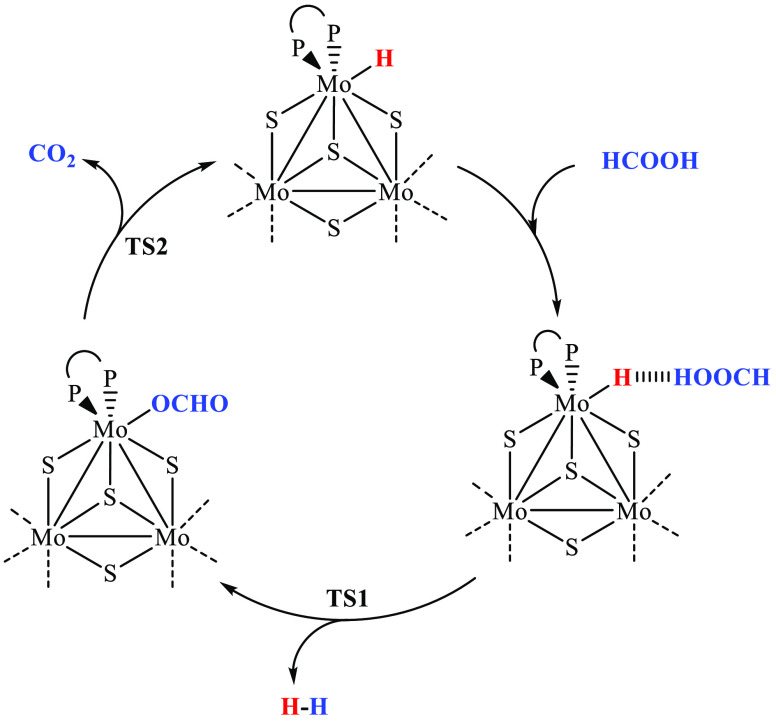
Proposed Catalytic Cycle for the Conversion of HCOOH to H_2_ and CO_2_ in the Presence of the Molybdenum Cluster

The results in the previous sections clearly show that [Mo_3_S_4_(OCHO)_3_(dmpe)_3_]^+^ is the main product of the reaction between [Mo_3_S_4_H_3_(dmpe)_3_]^+^ and FA. However, whereas at low temperatures the process ends at this point, catalytic formation of CO_2_ and H_2_ is observed at 100–120 °C. This indicates that CO_2_ is released from the coordinated formate ligands, with the resulting hydrides then being able to further react with FA. Scheme 3 schematically shows the catalytic cycle simplified to a single Mo center, with the same reactions being expected to take place at the three metal centers.

**Figure 7:**
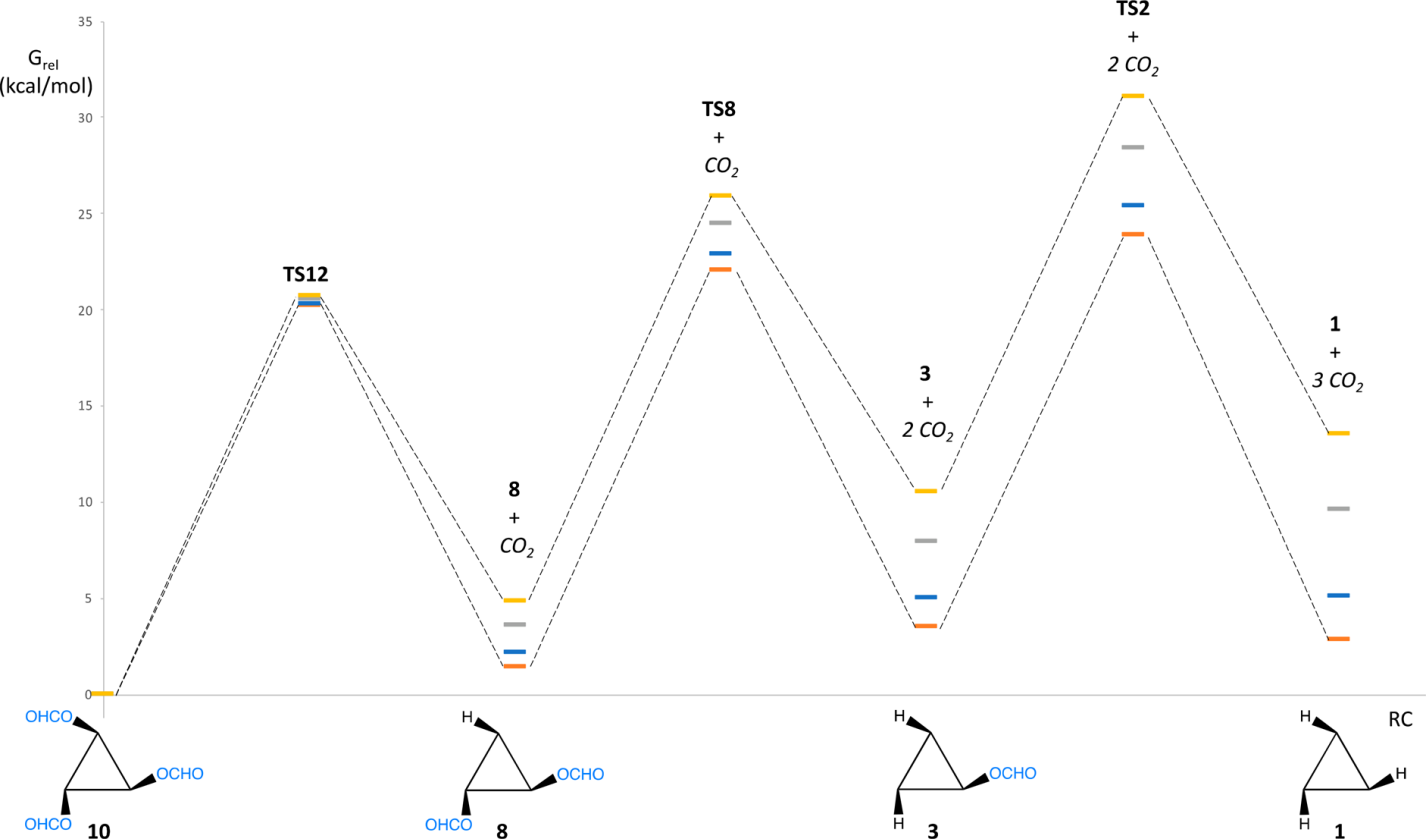
Free energy profile for the elimination of CO_2_ from [Mo_3_S_4_(OCHO)_3_(dmpe)_3_]^+^ (**10**) in PC solution at different temperatures. Color code: 25 °C (yellow), 60 °C (gray), 100 °C (blue), and 120 °C (orange).

At this point, we focused our efforts toward the isolation of the triformate cluster by reacting the hydride [Mo_3_S_4_H_3_(dmpe)_3_](BPh_4_) cluster salt with a buffer mixture of HCOOH:HCOONa in tetrahydrofuran at room temperature. Substitution of the hydrido ligands by formate occurs with a color change from brown-reddish to green. The green solid was characterized as [Mo_3_S_4_(OCHO)_3_(dmpe)_3_](BPh_4_) by NMR and Q-TOF mass spectrometry (Figures S8–S11, SI). The high resolution mass spectrum showed a peak centered at *m*/*z* = 1000.7817 with the isotopic pattern of the [Mo_3_S_4_(OCHO)_3_(dmpe)_3_]^+^ cluster cation. Thus, the role of the [Mo_3_S_4_(OCHO)_3_(dmpe)_3_]^+^ species as the resting state in the catalytic cycle could be confirmed by carrying out catalytic experiments. To our delight, the triformate cluster showed a similar activity to that of its precursor, catalyzing the FA dehydrogenation with a TON value analogous to the hydride cluster (Table S1 and Figure S12, SI). Next, we carried out DFT calculations on the elimination of CO_2_ from the triformate cluster and found that decarboxylation of each one of the three formate ligands to form the corresponding hydride occurs through a single transition state, with energy profiles at different temperatures shown in Figure 7 (see Table S3 for the *G*_rel_ values). Although the three steps are thermodynamically unfavored, the values of Δ*G*^0^ for a given step decrease significantly when the temperature is increased (the total Δ*G*^0^ decreases from 13.5 at 25 °C to 2.8 at 120 °C), in agreement with the experimental observation that CO_2_ is only formed at the highest temperatures used in the catalytic experiments. Three consecutive CO_2_ eliminations occur with close activation barriers, but the process is surely more complicated because the same cluster species ([Mo_3_S_4_H(OCHO)_2_(dmpe)_3_]^+^, [Mo_3_S_4_H_2_(OCHO)(dmpe)_3_]^+^, and [Mo_3_S_4_H_3_(dmpe)_3_]^+^) also participate in the formation of H_2_ upon reaction with FA, as seen in Scheme 2. The DFT-optimized structures of the reactant (**3**) and transition state (**TS2**), associated with the CO_2_ elimination at the former species, are included in Figure 5. These structures show that reaching **TS2** requires a large increase of more than 0.6 Å in the Mo–O bond distance, together with a decrease of more than 2 Å in the Mo–H interaction. Such changes indicate that the reaction occurs with a transition state in which there is a substantial degree of Mo–O bond breaking and Mo–H bond formation.

The species shown in Scheme 2 are involved in a complex network of elementary processes that include two possible processes for the terminal **1**, **5**, and **10** species, up to four processes for the **2**, **3**, **4**, **7**, **8**, and **9** intermediates and six processes for the complex **6**. Therefore, there are several possible pathways with close energy profiles for the overall catalytic reaction (see Figures S13 and S14, SI), so that the actual experimental pathway will depend not only on the magnitude of the different barriers but also on the experimental conditions.

## Conclusions

Dehydrogenation of formic acid has been tested for the first time using a cuboidal Mo_3_S_4_ cluster as catalyst in the absence of any additives. Compared to previous molybdenum homogeneous catalysts, the [Mo_3_S_4_H_3_(dmpe)_3_]^+^ hydride shows improved activity with a maximum TOF of 116 h^–1^ under optimized conditions and, more importantly, full selectivity toward CO_2_ and H_2_. Mechanistic investigations show that the reaction starts with the formation of dihydrogen-bonded species able to release H_2_, with the resulting formate ligands occupying the generated vacant sites at the Mo centers. This substitution reaction occurs in a single kinetic step, which can be interpreted in terms of statistical kinetics at the three metal centers. Reaction monitoring by mass spectrometry revealed the [Mo_3_S_4_(OCHO)_3_(dmpe)_3_]^+^ species as an active intermediate in the catalytic process. This triformate derivative was independently prepared and characterized, and tests on its catalytic activity showed a similar TOF to its hydride precursor. Notably, while the reaction at temperatures up to 60 °C ends at this triformate complex, catalytic formation of CO_2_ and H_2_ is only observed at 100–120 °C. This indicates that CO_2_ is released from the coordinated formate ligands in a process that results in the regeneration of the [Mo_3_S_4_H_3_(dmpe)_3_]^+^ hydride and therefore closes the catalytic cycle. DFT calculations fully agree with the experimental findings in that higher temperatures are needed for the β-hydride elimination that allows for the CO_2_ release and the recovery of the hydrido cluster. As shown by the computations, up to 10 forms of the cluster species can be involved in a complex network of elementary reactions. As a consequence, several possible pathways with close energy profiles for the overall catalytic process can be found, making apparent the complexity of the cluster catalysis process herein reported.

## Experimental Section

### Materials and Methods

All reactions were carried out under a nitrogen atmosphere using standard Schlenck techniques. Compound [Mo_3_S_4_H_3_(dmpe)_3_]Cl was prepared by following literature procedures.^28^ The remaining reactants were obtained from commercial sources and used as received. Solvents were purified by using a MBRAUN SPS-800 system. ^1^H, ^13^C{1H}, and ^31^P{^1^H} NMR spectra were recorded on a Bruker Avance III HD 400 MHz using CD_2_Cl_2_ as a solvent and referenced to the residual protons of the deuterated solvent or to 85% H_3_PO_4_. ESI-mass spectra were recorded using a Premier Q-TOF (quadrupole-hexapole-TOF) mass spectrometer with an orthogonal Z-spray electrospray source (Waters, Manchester, UK). Time-of-flight (TOF) mass spectra were acquired in the V-mode at a resolution of ca. 10 000 [full width at half-maximum (fwhm)]. Chemical identification of the cluster species was carried out by comparing the experimental and theoretical isotopic patern calculated from their elemental composition by using the MassLynx 4.1 program.^36^ The kinetics of reaction of the cluster with FA was studied using a Cary 50 Bio spectrophotometer provided with a thermostated multicell accessory. All of the experiments were carried out under pseudo-first-order conditions of acid excess. The reaction was monitored by following the spectral changes at a wide spectral range, and the data were analyzed using the program Specfit.^37^

### Computational Details

DFT calculations were run with Gaussian 09 (revision B.01).^38^ Geometry optimizations were carried out without symmetry restrictions at the BP86 level,^39^ with Mo and S atoms described using the SDD relativistic ECP and associated basis set,^40^ with added polarization functions for the latter (ζ = 0.503), and the remaining atoms described with the 6-31G(d,p) basis set.^41^ Solvent effects (propylene carbonate, Eps = 64.0, EpsInf = 2.019241) were included in these optimizations through the PCM method.^42^ Analytical frequency calculations were used to characterize each stationary point as a minimum or a transition state (TS). These calculations, carried out at four temperatures (see text) and 1 atm, also allowed obtainment of the thermal and entropic corrections required to calculate Gibbs energy values. Additionally, the Intrinsic Reaction Coordinate paths^43^ were followed along both directions of each TS vector to confirm the nature of the species connected by a given TS. The Gibbs energies discussed in the text were obtained by adding dispersion corrections via Grimme’s D3 parameter set (with Becke-Johnson damping) at the optimized species.^44^ Some test calculations at other theoretical levels are reported in Table S4, SI.

### Synthesis of [Mo_3_S_4_(HCOO)_3_(dmpe)_3_](BPh_4_)

A brown-redish solution of [Mo_3_S_4_H_3_(dmpe)_3_](BPh_4_) (0.030 g, 25,2 mmol) in THF (4 mL) was reacted with 300 μL of a HCOOH/HCOONa buffer solution (500 μL/200 mg) at room temperature for 15 h. The resulting reaction mixture was filtered under nitrogen to eliminate formate salts and the desired product was precipitated with diethyl ether as a green solid and separated by filtration to afford 0.028 g (84%) of [Mo_3_S_4_(HCOO)_3_(dmpe)_3_](BPh_4_).

^1^H NMR (400 MHz, CD_2_Cl_2_): δ 8.4 (s, 3H, *H*COO^–^), 2.9 (m, 4H, −C*H*_2_−), 2.3 (m, 6H, −C*H*_2_−), 2.1 (d, 10H, −C*H*_3_, −C*H*_2_−), 2.0 (d, 10H, −C*H*_3_, −C*H*_2_−), 1.4 (d, 9H, −C*H*_3_), 2.1 (d, 9H, −C*H*_3_). ^13^C{^1^H} NMR (100.4 MHz, CD_2_Cl_2_): 170.53 (s, H*C*O_2_^–^), 136.52, 126.18, 122.25 (s, Ar, BPh_4_^–^), 2.26 (m, −*C*H_2_−), 28.13–27.47 (m, −*C*H_2_−), 19.84 (d, −*C*H_3_), 14.42 (d, −*C*H_3_), 13.19 (d, −*C*H_3_), 13.14 ppm (d, −*C*H_3_). ^31^P{^1^H} NMR (161.9 MHz, CD_2_Cl_2_): δ 36.5 (dd, 3P), 17.4 (dd, 3P). Q-TOF-MS (20 V, CH_3_CN): *m*/*z* 1000.7817 [M^+^].

## Supporting Information Available

Details on materials and methods, catalytic protocol, additional information on catalytic data, synthesis and characterization of the [Mo_3_S_4_(OCHO)_3_(dmpe)_3_](BPh_4_) cluster, NMR and ESI-MS spectra, and kinetic and theoretical details.
