# Supplementary material for: Base-Free Catalytic Hydrogen Production from Formic Acid Mediated by a Cubane-Type Mo3S4 Cluster Hydride
Source: Inorg Chem. 2022 Oct 14;61(42):16730–9. doi: 10.1021/acs.inorgchem.2c02540 (PMC9690164; doi:10.1021/acs.inorgchem.2c02540)
Supplement: Supplementary file 1 — ic2c02540_si_001.pdf [file ic2c02540_si_001.pdf]

Supporting Information

Base-free catalytic hydrogen production from formic acid mediated by a cubane-type Mo_3S_4 cluster hydride

*Eva Guillamón,[a] Iván Sorribes,[a,b] Vicent S. Safont, [a] Andrés G. Algarra, [c] M. Jesús Fernández-Trujillo,[c] Elena Pedrajas,[c] Rosa Llusar,[*a] and Manuel G. Basallote[*c]*

[a] Departament de Química Física i Analítica, Universitat Jaume I, Av. Sos Baynat s/n, 12071 Castelló, Spain
[b] Current address: Instituto de Tecnología Química-Universitat Politècnica de València-Consejo Superior de Investigaciones Científicas (UPV-CSIC), 46022 Valencia, Spain.
[c] Departamento de Ciencia de los Materiales e Ingeniería Metalúrgica y Química Inorgánica, Instituto de Biomoléculas (INBIO), Facultad de Ciencias, Universidad de Cádiz, Apartado 40, Puerto Real, 11510 Cádiz, Spain.
E-mail: rosa.llusar@uji.es and manuel.basallote@uca.es

1. General details

2. Dehydrogenation of formic acid on manual burettes

3. H_2 and CO_2 molar volume, TON and TOF calculation

4. Gas chromatography spectra of the gas evolved from formic acid decomposition

5. Gas evolution monitoring for the recycling experiment

6. Reaction monitoring by ESI mass spectrometry and ^1H NMR

7. Kinetic experiments

8. Characterization of $[Mo_3S_4(HCO_2)_3(dmpe)_3]BPh_4$

9. Catalytic activity and gas evolution monitoring of $[Mo_3S_4(HCO_2)_3(dmpe)_3]BPh_4$ in the FA dehydrogenation

10. Computational details

1. General details

The $[Mo_3S_4H_3(dmpe)_3]BPh_4$ (dmpe = 1,2-bis(dimethylphosphine)ethane) cluster catalyst was prepared according to the published procedure.[1] Mass spectra were registered in a QTOF Premier instrument equipped with an orthogonal Z-spray-electrospray interface (Waters, Manchester, UK) operated in the V-mode at a resolution of ca. 10 000 (FWHM). The drying and cone gas was nitrogen set to flow rates of 300 and 30 L h^{-1}, respectively. A capillary voltage of 3.5 kV was used in the positive scan mode and the cone voltatge was set to Uc = 20 V. Chemical identification of the cluster species were carried out by comparing the experimental and theoretical isotopic pattern calculated from their elemental composition by using the MassLynx 4.1 program. NMR spectra were recorded on a Bruker Avance III HD 400 MHz spectrometer using CD_2Cl_2 as solvent. All other reagents were obtained from commercial sources and used as received.

2. Dehydrogenation of formic acid on manual burettes

Set-up used. Reaction was carried out in a vial provided with a valve system through which is connected to the gas burettes to measure the gas evolution. Once all reactants and solvents are introduced in the vial, this is placed in a reactor to control the temperature reaction.

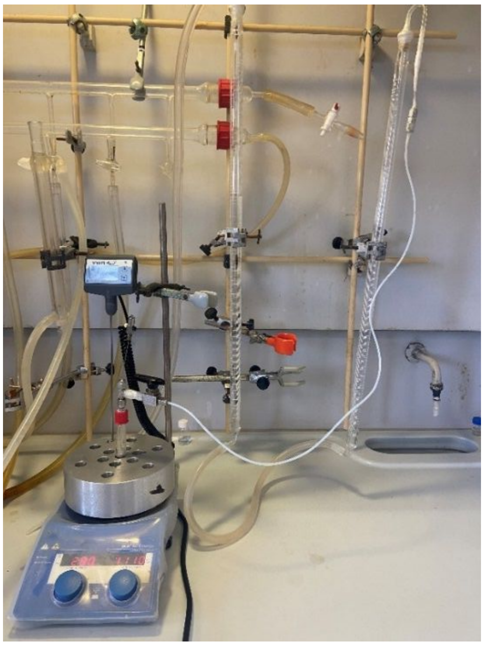

Procedure for the dehydrogenation of FA on manual burettes. Cluster catalyst (10 mg) is added to the vial which contains propylene carbonate (1,5 mL). Following, formic acid

(39 μL, 1 mmol) is added to the vial which is then closed and connected to the inverted water-filled burettes that were used to collect the released gas. After that, the vial is introduced in the reactor, preheated at 120ºC, and the valve is opened to the gas burettes system.

Gas chromatography analysis of the gas evolved. H_2 and CO_2 evolved gases were analyzed injecting a gas aliquot in a micro-GC with two columns (Molsieve 5A and PorePlot Q) and a TCD detector, whereas the absence of CO in the gaseous mixture was verify by using a gas chromatograph (Agilent 7890A) equipped with Carboxene 1010 column and a TCD detector (CO detection limit ~10 ng·ml^{-1}).

3. H_2 and CO_2 molar volume, TON and TOF calculation

Molar volume of H_2 and CO_2. Molar volumes can be easily calculated from the Van der Waals equation as follows:

$$V_{m,H_2} = \frac{R\cdot T}{p} + b - \frac{a}{R\cdot T} \qquad (1)$$

and

$$V_{m,CO_2} = \frac{R\cdot T}{p} + b - \frac{a}{R\cdot T} \qquad (2)$$

where R = 8,3145 m^3·Pa·mol^{-1}·K^{-1}; T = 273,15 + room temperature (ºC) K; p = 101325 Pa; a_{H_2} = 24,9·10^{-3} Pa·m^6·mol^{-2}; b_{H_2} = 26,7·10^{-6} m^3·mol^{-1}; a_{CO_2} = 36,5·10^{-2} Pa·m^6·mol^{-2}; b_{CO_2} = 42,7·10^{-6} m^3·mol^{-1}

Turnover number calculation. Gas evolution was corrected with the blank volume taking into account that H_2 : CO_2 ratio is 1 : 1 according to GC measurements. The blank volume was measured without catalyst. Then, the turnover number, TON, is calculated as follows:

$$TON = \frac{\frac{V_{obs} - V_{blank}}{V_{m,H_2} + V_{m,CO_2}}}{n_{cat}} \qquad (3)$$

Turnover number frequency calculation. The turnover number frequency is calculated according to the following equation and linked to the temporal units (s, min, h)

$$TOF = \frac{TON}{time} \qquad (4)$$

4. Gas chromatography spectra of the gas evolved from formic acid decomposition

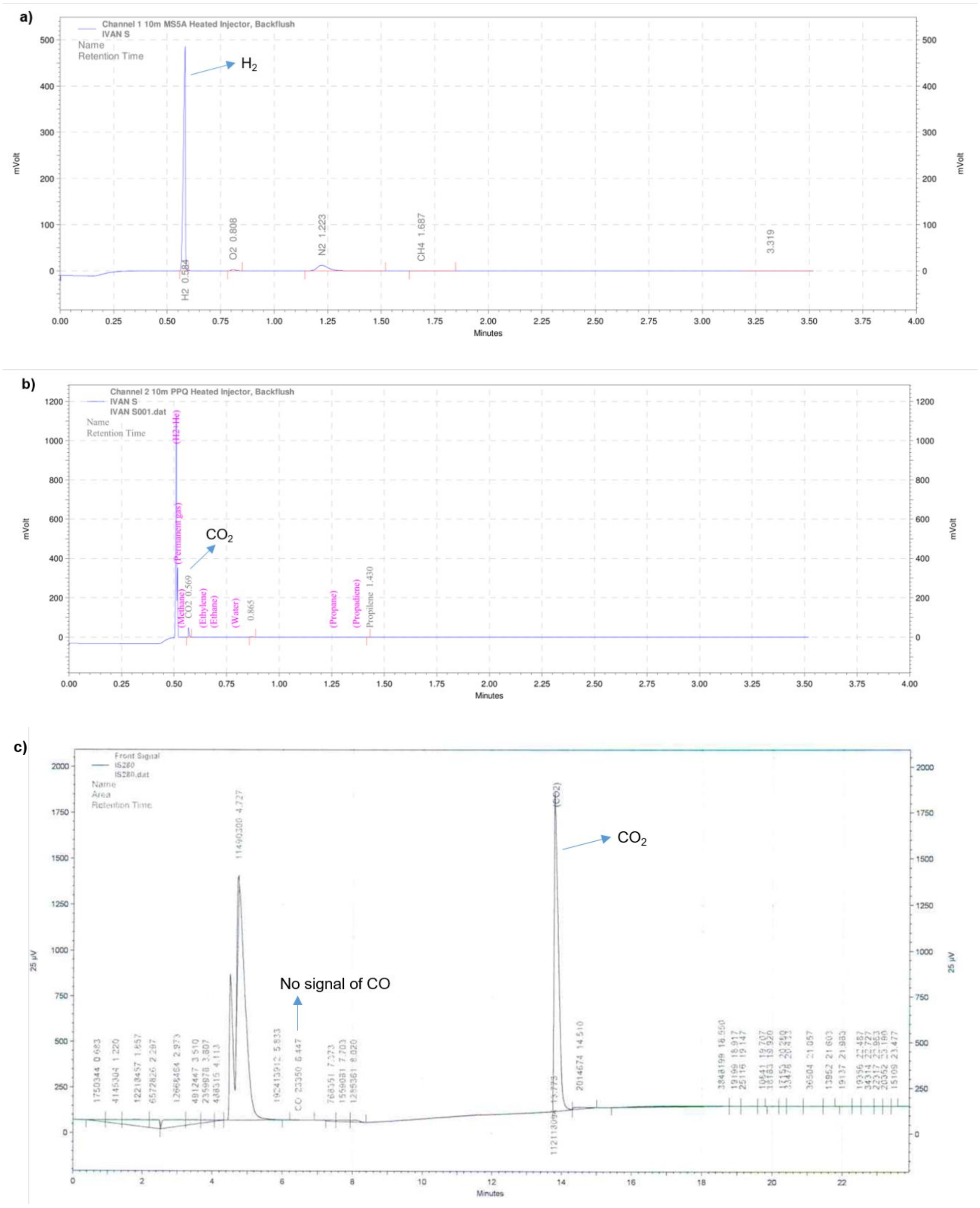

Figure S1. Micro-GC (**a-b**) and GC (**c**) spectra of the gas evolved from the formic acid decomposition reaction at 120 ºC in the presence of the cluster catalyst $[Mo_3S_4H_3(dmpe)_3](BPh_4)$.

5. Gas evolution monitoring for the recycling experiments

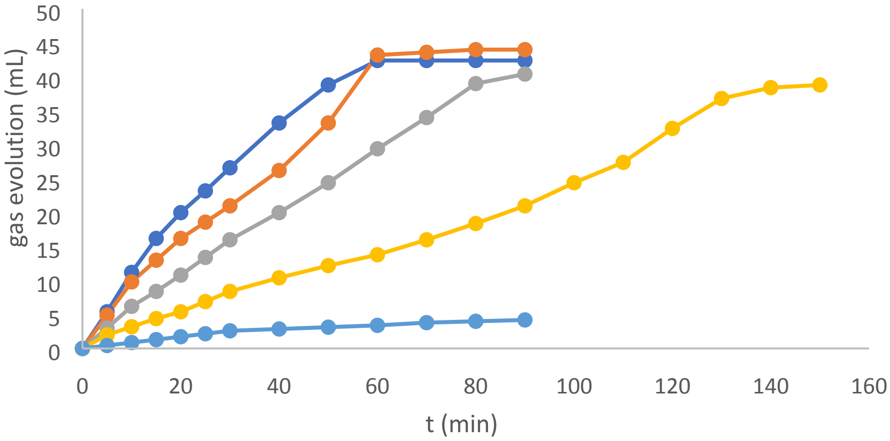

Figure S2. Sequential additions of formic acid in the presence of the $[Mo_3S_4H_3(dmpe)_3]^+$ catalyst. Reaction conditions: HCOOH (1 mmol), $[Mo_3S_4H_3(dmpe)_3](BPh_4)$ (8,64 µmol) and 1,5 mL of propylene carbonate were used. HCOOH (1 mmol) was added after cooling the reaction mixture to room temperature: run 1 (blue), run 2 (orange), run 3 (grey), run 4 (yellow) and run 5 (green).

6. Reaction monitoring by ESI mass spectrometry and ^{1}H NMR

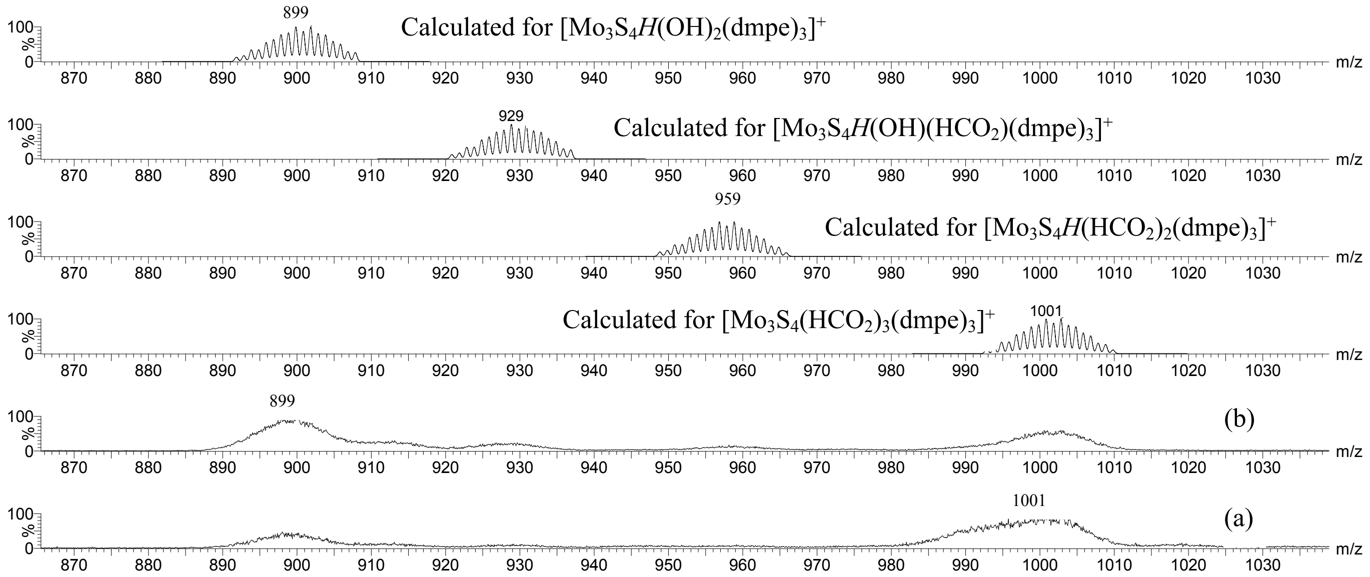

Figure S3. Mass spectra of the reaction mixture after 20 min (a) and at the end of the reaction (b).

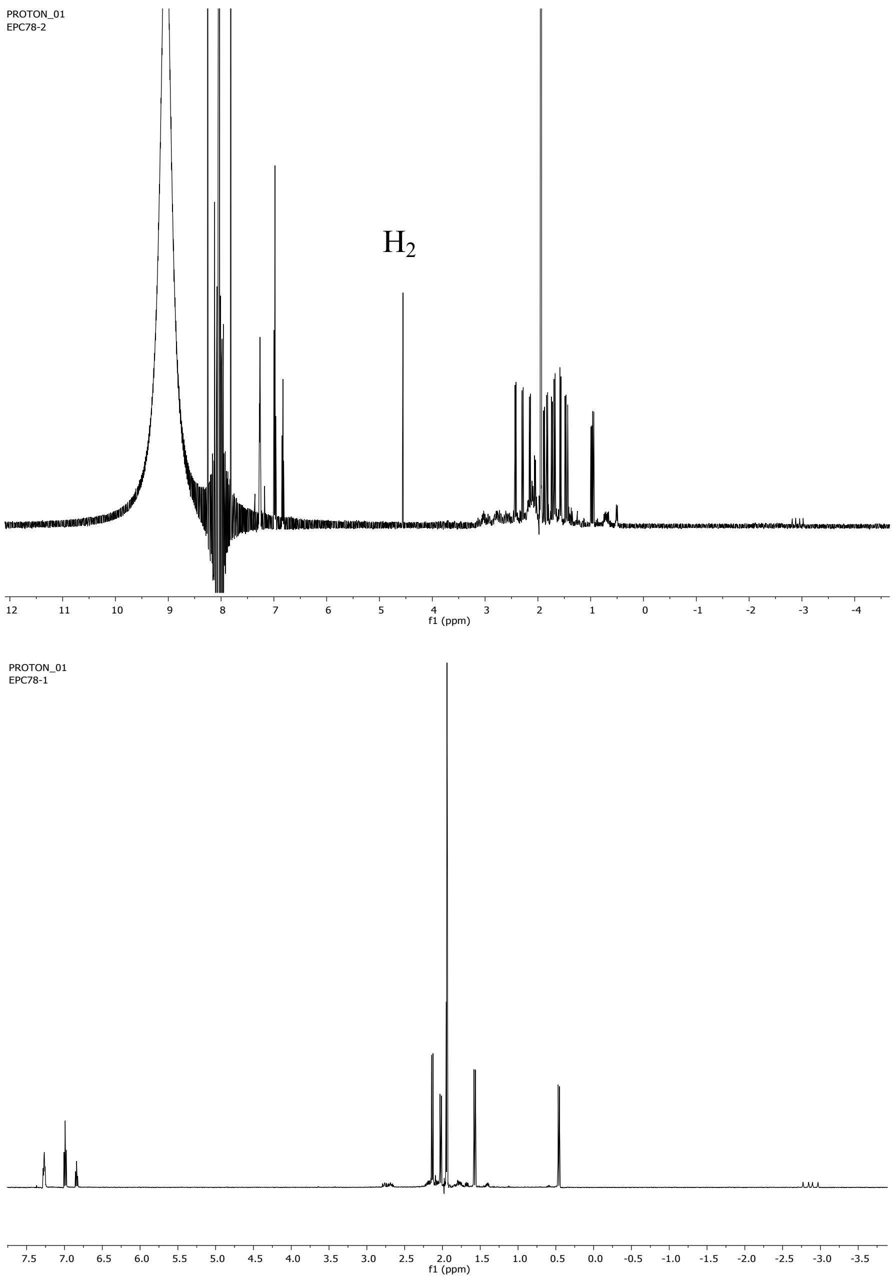

Figure S4. ^{1}H NMR spectra recorded before (bottom) and after (top, c.a. 15 min reaction time) addition of FA to a CD_3CN solution of the cluster at room temperature.

7. Kinetic experiments

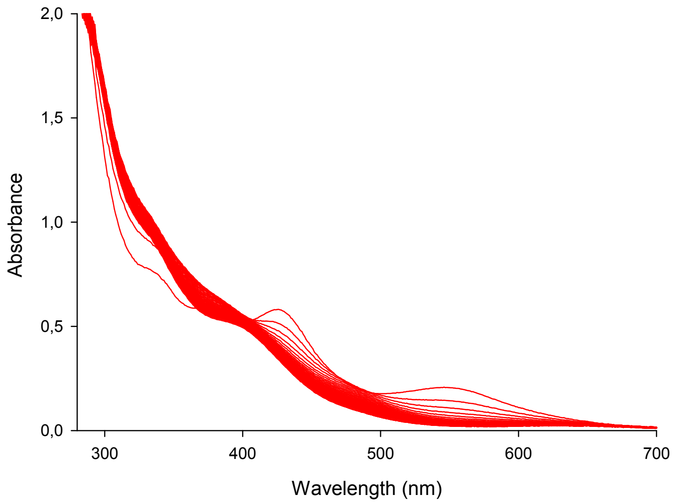

Figure S5. Spectral changes observed during 2650 min for the reaction of the $[Mo_3S_4H_3(dmpe)_3](BPh_4)$ cluster (1.5×10^{-4} M) and HCOOH (0.006 M) in propylene carbonate.

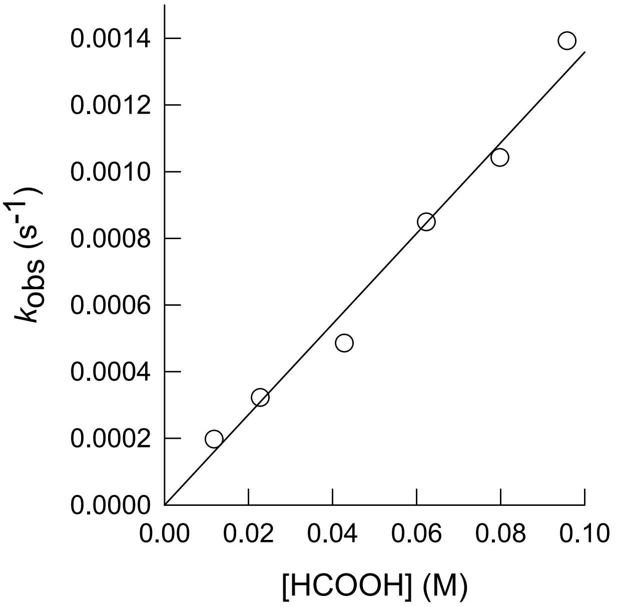

Figure S6. Plot of the dependence of the rate constants in the reaction of cluster $[Mo_3S_4H_3(dmpe)_3]^+$ with HCOOH in propylene carbonate solution at 60ºC.

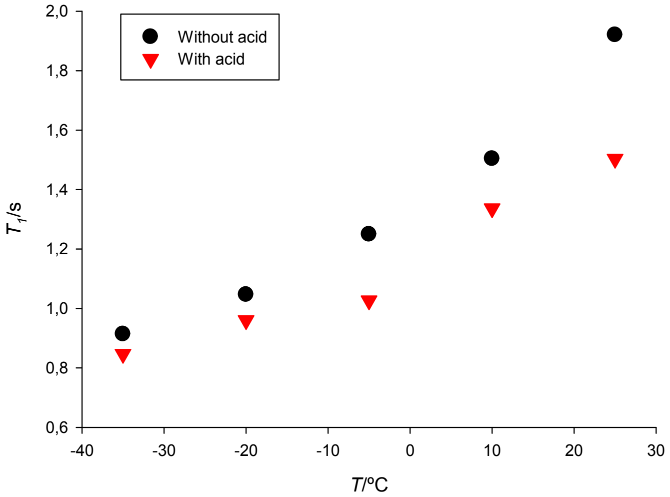

Figure S7. Temperature dependence of the T_1 value for the hydride signal of complex $[Mo_3S_4H_3(dmpe)_3]^+$ in CH_3CN solution. The circles correspond to the data for the complex alone and the triangles for the complex with an excess of HCOOH (44 equivalents).

8. Characterization of $[Mo_3S_4(HCO_2)_3(dmpe)_3]BPh_4$

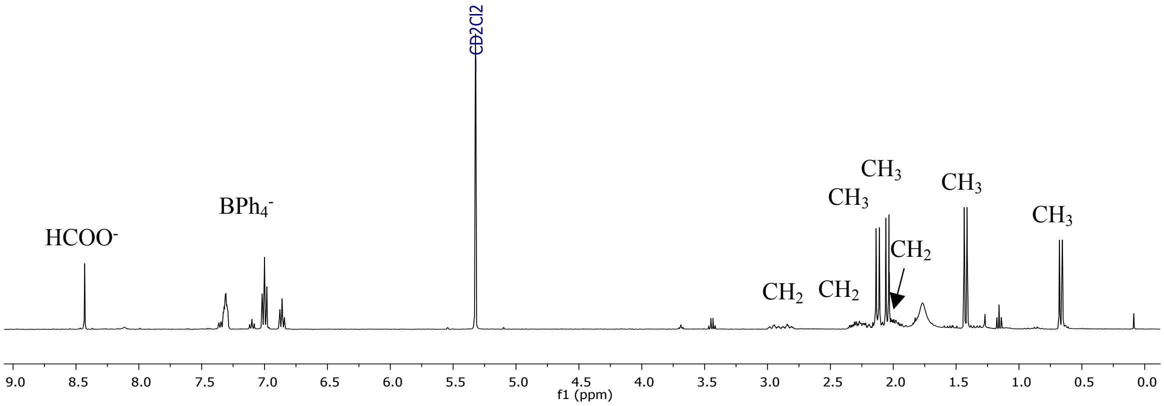

Figure S8. ^1H NMR spectrum of $[Mo_3S_4(OCHO)_3(dmpe)_3]BPh_4$ in CD_2Cl_2.

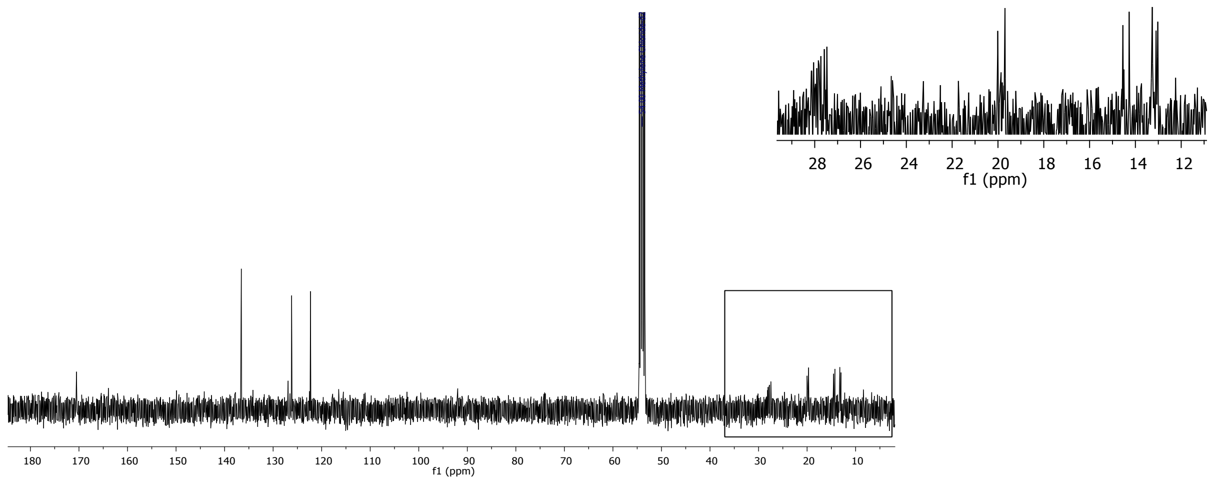

Figure S9. $^{13}C\{^1H\}$ NMR spectrum of $[Mo_3S_4(OCHO)_3(dmpe)_3]BPh_4$ in CD_2Cl_2.

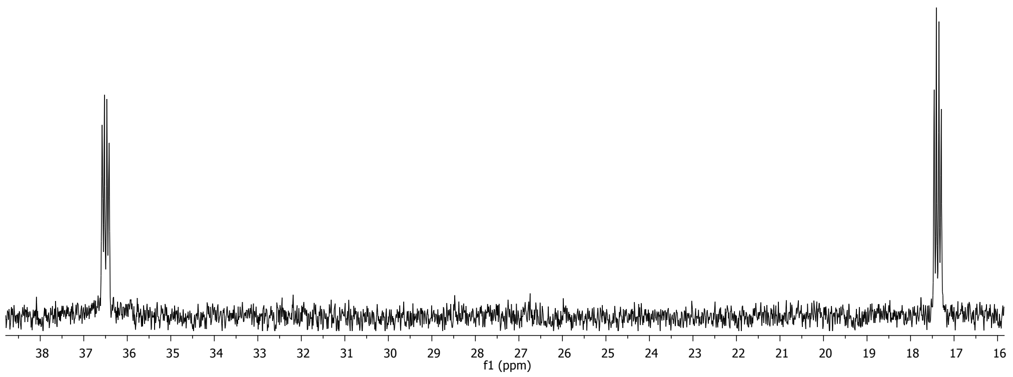

Figure S10. $^{31}P\{^1H\}$ NMR spectrum of $[Mo_3S_4(OCHO)_3(dmpe)_3]BPh_4$ in CD_2Cl_2.

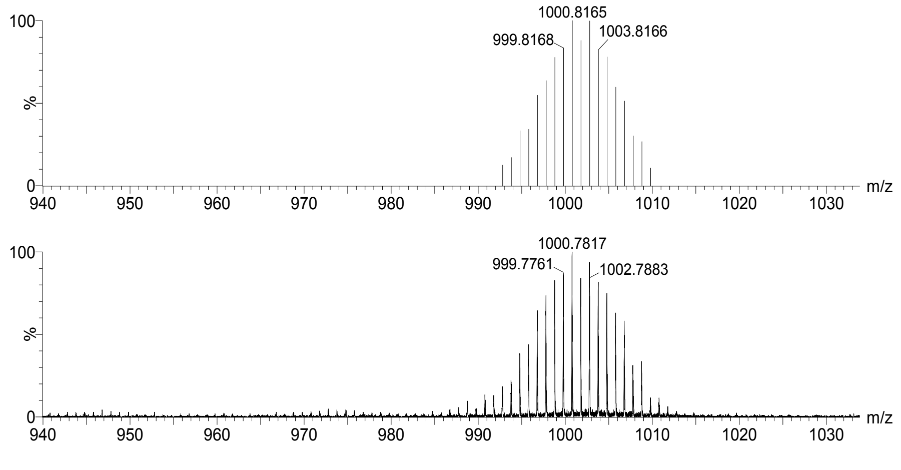

Figure S11. Experimental Q-TOF mass spectrum of $[Mo_3S_4(OCHO)_3(dmpe)_3]^+$ in CH_3CN (bottom) and calculated (up).

9. Catalytic activity and gas evolution monitoring of $[Mo_3S_4(HCO_2)_3(dmpe)_3]BPh_4$ in the FA dehydrogenation

Table S1. FA dehydrogenation in the presence of different catalysts.[a]

Catalyst	Gas Volume (mL)[b]	Time (min)[c]	Conversion (%)	TON	TOF (h^{-1})
$[Mo_3S_4H_3(dmpe)_3]^{+(d)}$	42.8	70	88	116	116
$[Mo_3S_4(HCO_2)_3(dmpe)_3]^{+(e)}$	40.8	90	83	110	82

[a] 1,5 mL of propylene carbonate and 1mmol of HCOOH were used. [b] $H_2 + CO_2$ volume monitored with manual burettes and corrected by the blank volume (0,4 mL). Experiments were performed at least twice (Standard deviation < 10%). [c] Time required to completeness. [d] 7,57 μmol of the catalyst were used. [e] 8,64 μmol of catalyst were used.

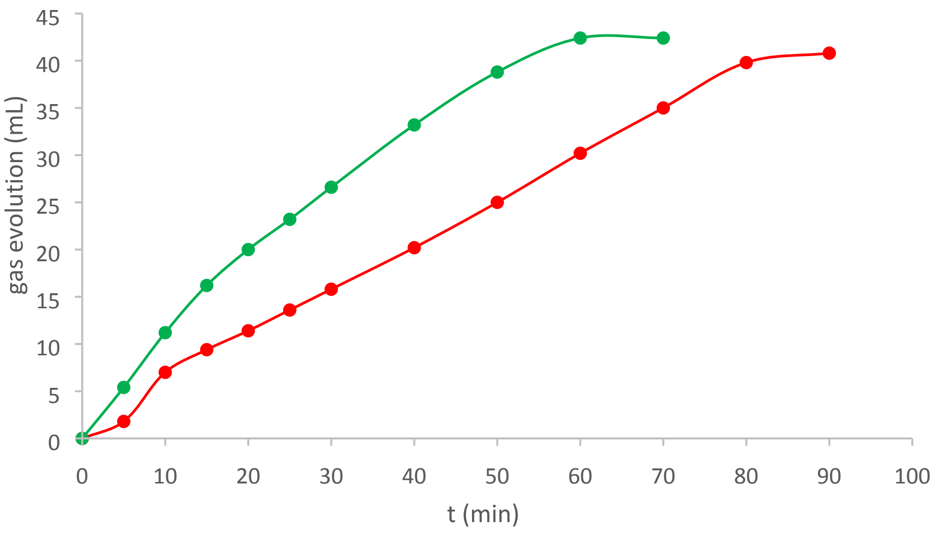

Figure S12. Catalytic dehydrogenation of formic acid in the presence of the $[Mo_3S_4H_3(dmpe)_3]^+$ (green) and $[Mo_3S_4(HCO_2)_3(dmpe)_3]^+$ (red) clusters. Reaction conditions: HCOOH (1 mmol), 1,5 mL of propylene carbonate and $[Mo_3S_4H_3(dmpe)_3](BPh_4)$ (8,64 μmol) or $[Mo_3S_4(HCO_2)_3(dmpe)_3](BPh_4)$ (7,57 μmol) were used. T = 120ºC

10. Computational details

Table S2. Total Gibbs free energies (Hartrees/particle) of the indicated species at the shown temperatures. The values include the solvent effect as well as the Grimme correction.

**Species**	**G at 298 K**	**G at 333 K**	**G at 373 K**	**G at 393 K**
HCOOH	-189.763868	-189.767220	-189.771133	-189.773121
CO_2	-188.599701	-188.602591	-188.605962	-188.607673
H_2	-1.178296	-1.180056	-1.182118	-1.183167
**1**	-3010.887989	-3010.904469	-3010.924802	-3010.935551
**2**	-3200.652817	-3200.670396	-3200.692087	-3200.703554
**3**	-3199.492432	-3199.509624	-3199.530849	-3199.542073
**4**	-3390.419419	-3390.438265	-3390.461509	-3390.473791
**5**	-3580.186270	-3580.206400	-3580.231213	-3580.244319
**6**	-3389.259612	-3389.278073	-3389.300852	-3389.312893
**7**	-3579.025938	-3579.045757	-3579.070192	-3579.083099
**8**	-3388.101171	-3388.119160	-3388.141373	-3388.153120
**9**	-3577.867883	-3577.887214	-3577.911067	-3577.923674
**10**	-3576.708644	-3576.727486	-3576.750754	-3576.763057
**TS1**	-3200.614443	-3200.631683	-3200.652978	-3200.664245
**TS2**	-3199.459729	-3199.477040	-3199.498391	-3199.509674
**TS3**	-3390.383909	-3390.402509	-3390.425462	-3390.437597
**TS4**	-3389.227173	-3389.245795	-3389.268746	-3389.280868
**TS5**	-3580.147523	-3580.167520	-3580.192177	-3580.205205
**TS6**	-3579.992740	-3579.012560	-3579.036984	-3579.049882
**TS7**	-3389.226423	-3389.244759	-3389.267388	-3389.279352
**TS8**	-3388.067692	-3388.085914	-3388.108385	-3388.120258
**TS9**	-3579.990550	-3579.010130	-3579.034302	-3579.047083
**TS10**	-3577.833740	-3577.853141	-3577.877064	-3577.889702
**TS11**	-3577.831458	-3577.850492	-3577.873996	-3577.886426
**TS12**	-3576.675651	-3576.694792	-3576.718394	-3576.730861

With the data in Table S2 several cyclic routes can be traced with participation of three HCOOH molecules involving one, two or three metal centers:

$$\mathbf{1} + 3\ \mathrm{HCOOH} \rightarrow 3\ \mathrm{CO_2} + 3\ \mathrm{H_2} + \mathbf{1} \qquad (5)$$

Some of these routes are presented in the following figures:

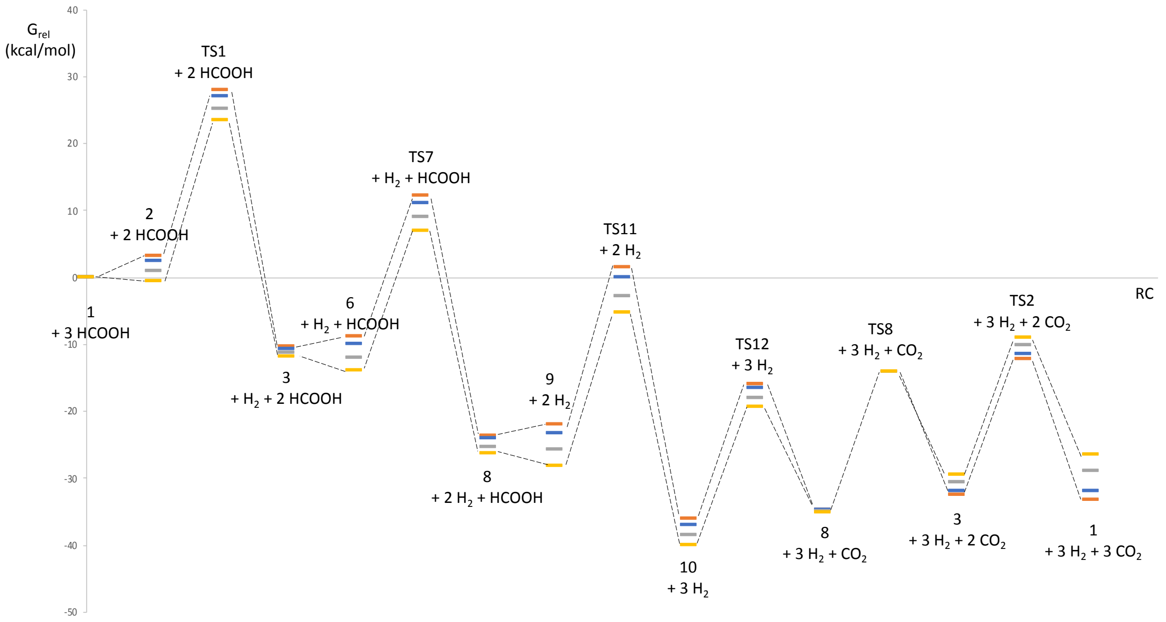

Figure S13. Free energy profile for the $[Mo_3S_4H_3(dmpe)_3]^+$ catalyzed decomposition of formic acid (3 HCOOH → 3 CO_2 + 3 H_2) assuming that all the H_2 eliminations occur first and the three CO_2 eliminations later. Species 1, 3, 8 and 10 correspond to $[Mo_3S_4H_3(dmpe)_3]^+$, $[Mo_3S_4H_2(OCHO)(dmpe)_3]^+$, $[Mo_3S_4H(OCHO)_2(dmpe)_3]^+$ and $[Mo_3S_4(OCHO)_3(dmpe)_3]^+$, respectively, and species 2, 6 and 9 to their corresponding dihydrogen-bonded adducts with FA. Color code for temperatures: 25ºC (yellow), 60ºC (grey), 100ºC (blue) and 120ºC (orange).

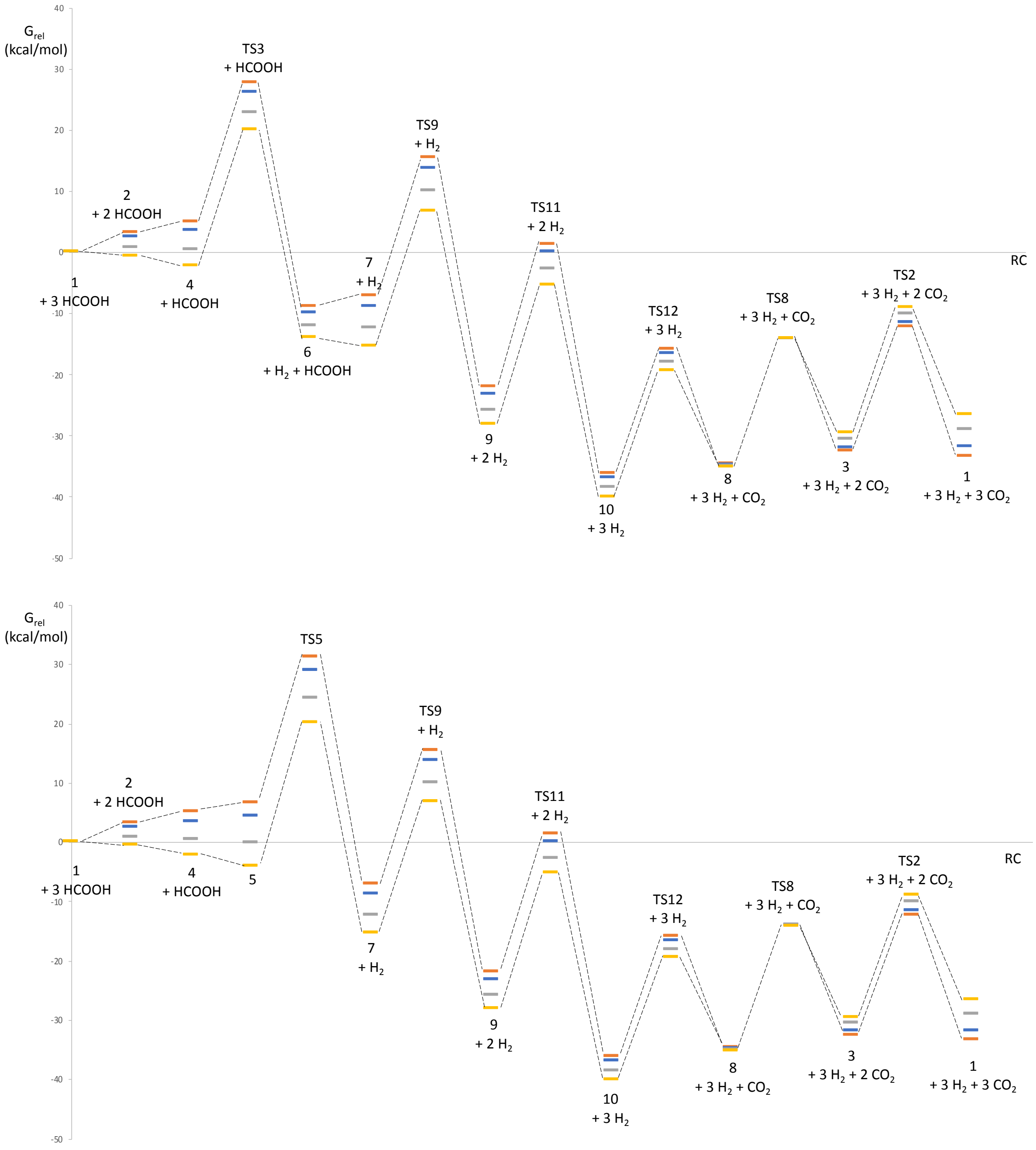

Figure S14. Two free energy profiles for the $[Mo_3S_4H_3(dmpe)_3]^+$ catalyzed decomposition of formic acid (3 HCOOH → 3 CO_2 + 3 H_2) assuming different combinations for the ordering of the H_2 and CO_2 elimination steps. Upper figure: pathway through TS3; bottom figure: pathway through TS5. Color code for temperatures: 25ºC (yellow), 60ºC (grey), 100ºC (blue) and 120ºC (orange).

Table S3. Relative Gibbs free energies (kcal/mol) of the species appearing in Figures 6 (the first 10 entries) and 7 (the last 7 entries) of the manuscript, at the shown temperatures. Note that the reference corresponds to the reactants (**1** + 3 HCOOH) in the Figure 6 case, and to **10** in the Figure 7 case. The values include the solvent effect as well as the Grimme correction.

**Species**	**25 ºC**	**60 ºC**	**100 ºC**	**120 ºC**
**1**+3HCOOH	0.00	0.00	0.00	0.00
**2** + 2 HCOOH	-0.60	0.81	2.41	3.21
**4** + HCOOH	-2.32	0.40	3.49	5.02
**TS3** + HCOOH	19.96	22.84	26.11	27.73
**6** + H_2 + HCOOH	-13.92	-12.06	-9.98	-8.95
**7** + H_2	-15.46	-12.35	-8.85	-7.12
**TS9** + H_2	6.74	10.00	13.67	15.48
**9** + 2 H_2	-28.16	-25.85	-23.28	-22.02
**TS11** + 2 H_2	-5.31	-2.81	-0.02	1.35
**10** + 3 H_2	-40.12	-38.61	-36.96	-36.17
**10**	0.00	0.00	0.00	0.00
**TS12**	20.70	20.52	20.30	20.20
**8** + CO_2	4.87	3.60	2.14	1.42
**TS8** + CO_2	25.88	24.46	22.84	22.04
**3** + 2 CO_2	10.55	7.96	5.00	3.54
**TS2** + 2 CO_2	31.07	28.41	25.37	23.87
**1** + 3 CO_2	13.52	9.57	5.06	2.81

Table S4.

Single point calculations in PC solvent for the process 1 + HCOOH → 2

wB97XD/Def2QZVP(Mo)-Def2TZVP//BP86/BS1	No convergence
B3LYP/Def2QZVP(Mo)-Def2TZVP//BP86/BS1	No convergence
M06/Def2QZVP(Mo)-Def2TZVP//BP86/BS1	No convergence

Single point calculations in PC solvent for the process 1 + HCOOH → 2 via TS1, with corrections for G and thermal (BP86/BS1) plus D3-Grimme correction (single point on the BP86/BS1 geometry)

Level	*ΔG (at 298) (kcal/mol)*	*ΔG# (at 298) (kcal/mol)*
wB97XD/Def2QZVP//BP86/BS1	0.8	32.9

Optimizations in PC solvent for the process 1 + HCOOH → 2 via TS1

Level	*ΔG (at 298) (kcal/mol)*	*ΔG# (at 298) (kcal/mol)*
BP86/BS1	-0.6	23.5
B3LYP/BS1	-0.2	29.8
PBE0/BS1	1.4	33.7
BP86/Def2TZVP	3.2	-
PBE0-D3/BS1*	1.0	-

Optimizations in Gas Phase for the process 1 + HCOOH → 2

Level	*ΔG (at 298) (kcal/mol)*
BP86/BS1	-5.0
B3LYP/BS1	-4.8
wB97XD/BS1	-2.1
M06/BS1	-1.4
BP86/Def2TZVP	0.7
BP86-D3/BS1*	-6.4
B3LYP-D3/BS1*	-5.4
PBE0-D3/BS1*	-3.4
BP86-D3/Def2TZVP*	-0.9
PBE0-D3/Def2TZVP*	+1.7

** Calculations done with Gaussian16, at levels not available with Gaussian09*

Cartesian coordinates of the reported stationary points. E values obtained optimizing each point in PC solvent (PCM) at the BP86/BS1 theoretical level.

1

E=-3011.131698 Hartrees/particle

Mo	-1.090921	-1.198372	-0.005078
Mo	-0.512540	1.553243	-0.021247
Mo	1.586599	-0.322931	-0.003334
S	0.694423	-2.069361	-1.325704
S	-2.176682	0.437601	-1.316475
S	-0.008232	0.017520	1.758099
S	1.470196	1.674539	-1.302751
P	-2.653583	-2.814339	-1.137685
P	-0.867608	-3.240346	1.533076
P	3.764485	-0.881817	-1.138483
P	3.257315	0.822452	1.566427
P	-1.141057	3.701863	-1.167393
P	-2.360441	2.410383	1.540204
H	-2.385726	-1.381697	1.150775
H	-0.007734	2.771067	1.121791
H	2.356275	-1.390337	1.143218
C	0.039409	5.112942	-0.974475
H	0.173454	5.339146	0.094701
H	1.015597	4.821142	-1.392886
H	-0.334173	6.007714	-1.499047
C	-4.019135	1.585464	1.521032
H	-4.431956	1.603471	0.500499
H	-3.895501	0.533092	1.821208
H	-4.710315	2.095114	2.212508
C	-1.990540	2.523397	3.345497
H	-1.864698	1.509065	3.755707
H	-1.051738	3.080625	3.488266
H	-2.811066	3.032973	3.876935
C	-1.456842	3.681954	-2.988825
H	-0.523968	3.418779	-3.512299
H	-2.215692	2.917765	-3.218309
H	-1.804546	4.668785	-3.336492
C	-2.751496	4.179504	1.075489
H	-1.983690	4.814293	1.551990
H	-3.727941	4.462377	1.505843
C	-2.736161	4.360390	-0.450376
H	-3.557915	3.793040	-0.922778
H	-2.861675	5.420608	-0.732723
C	4.366550	-2.619569	-0.939435
H	4.469807	-2.852886	0.131594
H	3.623697	-3.306810	-1.374070
H	5.336263	-2.755480	-1.445792
C	3.925506	-0.599017	-2.958086
H	3.221196	-1.259024	-3.489210
H	3.668457	0.446975	-3.187023

H	4.952564	-0.812687	-3.297862
C	3.151072	0.457587	3.373238
H	2.224878	0.895470	3.777250
H	3.121467	-0.632782	3.522146
H	4.017777	0.883369	3.905288
C	3.405937	2.669122	1.542281
H	3.604133	3.012238	0.515169
H	2.451705	3.108151	1.872915
H	4.218860	2.996858	2.211105
C	4.980143	0.249418	1.116912
H	5.127690	-0.735836	1.593590
H	5.719252	0.942376	1.555742
C	5.141757	0.147675	-0.408327
H	5.077810	1.146166	-0.877070
H	6.119606	-0.285697	-0.683136
C	0.668481	-4.273255	1.445458
H	0.823900	-4.620184	0.411956
H	1.531347	-3.649922	1.728998
H	0.588349	-5.138672	2.123778
C	-1.084919	-2.999664	3.350854
H	-0.248786	-2.398905	3.741438
H	-2.025443	-2.457873	3.535337
H	-1.107971	-3.972260	3.869786
C	-2.226432	-4.449942	1.101491
H	-3.144426	-4.095381	1.603013
H	-1.980558	-5.439878	1.523885
C	-4.461266	-2.483991	-0.927579
H	-4.712913	-2.483157	0.144172
H	-4.691682	-1.488221	-1.338045
H	-5.061592	-3.247204	-1.449603
C	-2.429204	-4.525342	-0.420597
H	-1.541421	-4.961868	-0.912004
H	-3.296473	-5.156898	-0.682052
C	-2.496893	-3.085255	-2.959802
H	-2.724183	-2.143777	-3.484668
H	-1.462479	-3.379844	-3.196330
H	-3.193683	-3.869685	-3.298946

HCOOH
E=-189.769919 Hartrees/particle

H	-0.089732	0.000000	-0.055712
C	0.038756	0.000000	1.045705
O	1.109182	0.000000	1.632215
O	-1.178327	0.000000	1.636018
H	-1.025005	0.000000	2.609040

2
E=-3200.911741 Hartrees/particle

Mo	-1.175581	-1.176830	-0.065302
Mo	-0.582016	1.584657	-0.060861

Mo	1.490318	-0.313610	0.024427
S	0.622359	-2.015867	-1.367262
S	-2.218517	0.473758	-1.395042
S	-0.158221	0.037805	1.733840
S	1.440328	1.676830	-1.276444
P	-2.695917	-2.796789	-1.262913
P	-0.889939	-3.293134	1.399012
P	3.697913	-0.889550	-1.049982
P	3.125317	0.803209	1.649516
P	-1.061071	3.758696	-1.253090
P	-2.468248	2.553033	1.394058
H	-2.518448	-1.395549	1.059446
H	-0.096539	2.785790	1.107000
H	2.209852	-1.397280	1.190931
C	0.225634	5.075839	-1.078470
H	0.378526	5.301443	-0.011704
H	1.175975	4.707192	-1.495308
H	-0.083029	5.990767	-1.610371
C	-4.205368	1.936625	1.196248
H	-4.493386	1.968487	0.134134
H	-4.269625	0.895350	1.543652
H	-4.897537	2.558209	1.788152
C	-2.237870	2.571136	3.225500
H	-2.243976	1.537437	3.604569
H	-1.263446	3.027211	3.459764
H	-3.043732	3.144440	3.712666
C	-1.371211	3.722489	-3.074844
H	-0.467766	3.355970	-3.587415
H	-2.201740	3.031800	-3.289105
H	-1.619463	4.729847	-3.448015
C	-2.642777	4.364596	0.967378
H	-1.811275	4.898688	1.460027
H	-3.585293	4.748409	1.395428
C	-2.595640	4.560418	-0.555497
H	-3.463568	4.078147	-1.039713
H	-2.615530	5.629890	-0.828777
C	4.264521	-2.639879	-0.862094
H	4.329795	-2.894051	0.207135
H	3.525092	-3.306392	-1.333287
H	5.247541	-2.782858	-1.340110
C	3.919646	-0.579966	-2.858789
H	3.227164	-1.224861	-3.422984
H	3.680315	0.471927	-3.079900
H	4.955133	-0.799356	-3.167586
C	2.948628	0.438590	3.450480
H	2.007570	0.875513	3.819657
H	2.914040	-0.652063	3.596592
H	3.794808	0.863335	4.015372
C	3.302046	2.647031	1.626943
H	3.542260	2.984611	0.606892

H	2.343345	3.101190	1.922228
H	4.095242	2.964313	2.323862
C	4.850924	0.199651	1.259606
H	4.960230	-0.792013	1.733092
H	5.587126	0.874313	1.730405
C	5.065555	0.107814	-0.259558
H	5.037075	1.111629	-0.720331
H	6.044695	-0.340718	-0.503303
C	0.632188	-4.325813	1.186385
H	0.740544	-4.626991	0.133383
H	1.511393	-3.721986	1.460805
H	0.571242	-5.219910	1.828676
C	-0.986115	-3.137014	3.237142
H	-0.090816	-2.603569	3.593940
H	-1.875680	-2.557944	3.525346
H	-1.024990	-4.134473	3.705162
C	-2.273426	-4.468305	0.962163
H	-3.185712	-4.102671	1.467067
H	-2.041017	-5.469675	1.365532
C	-4.507989	-2.480385	-1.095692
H	-4.786546	-2.523626	-0.030478
H	-4.732761	-1.473820	-1.482796
H	-5.084051	-3.230167	-1.663180
C	-2.478226	-4.517455	-0.562356
H	-1.599429	-4.960452	-1.063998
H	-3.354088	-5.134956	-0.828977
C	-2.476876	-3.035966	-3.082551
H	-2.713385	-2.092520	-3.599854
H	-1.429094	-3.300954	-3.293622
H	-3.144096	-3.831770	-3.453221
H	-3.380129	-1.225229	2.228070
O	-3.918096	-1.066655	3.075248
C	-4.924356	-1.958183	3.146384
H	-5.522008	-1.781873	4.063722
O	-5.160801	-2.843065	2.332192

TS1
E=-3200.861386 Hartrees/particle

Mo	0.014693	0.004415	-0.118027
Mo	-0.088579	-0.063855	2.676931
Mo	2.515416	-0.042060	1.473125
S	1.685412	1.609937	-0.253491
S	-1.704084	0.873003	1.220737
S	0.857699	-1.797682	1.282109
S	1.494497	1.595241	2.990147
P	-1.290763	1.177128	-1.932285
P	0.833079	-1.256288	-2.226243
P	4.626627	1.339909	1.606718
P	3.464514	-0.756426	3.863787
P	-1.372060	1.128464	4.507115

P	-1.836756	-1.828765	3.327316
H	-1.119938	-1.159992	-0.785147
H	0.431588	-1.095587	3.987763
H	3.088454	-0.297028	-0.223172
H	5.005848	-3.353066	0.721074
C	4.331836	-2.498386	0.528688
O	4.014193	-1.761348	1.516104
O	3.930271	-2.307692	-0.672324
H	3.342758	-1.191879	-0.457205
C	5.949749	0.896043	0.388490
H	6.226192	-0.163548	0.500081
H	5.569666	1.055537	-0.632878
H	6.843819	1.521141	0.548214
C	4.518406	3.174923	1.429068
H	4.162502	3.417162	0.415360
H	3.794025	3.568654	2.158834
H	5.504876	3.638478	1.595318
C	2.938236	0.032673	5.450503
H	3.033079	1.127173	5.401396
H	1.885199	-0.218503	5.639052
H	3.569282	-0.359170	6.265800
C	3.454433	-2.542809	4.351078
H	2.415266	-2.838390	4.565873
H	3.844491	-3.156531	3.528436
H	4.068146	-2.694105	5.254659
C	5.282974	-0.302180	3.816581
H	5.793353	-1.042974	3.179921
H	5.699150	-0.387910	4.835768
C	5.467322	1.122881	3.266858
H	5.010972	1.863318	3.948182
H	6.536389	1.382750	3.171187
C	2.389536	-0.762807	-3.121047
H	2.488238	0.334085	-3.111196
H	3.271000	-1.197620	-2.630513
H	2.352511	-1.123838	-4.162459
C	-1.159274	3.016643	-2.070633
H	-1.568144	3.476500	-1.156786
H	-0.099452	3.300120	-2.162642
H	-1.720102	3.383429	-2.946352
C	0.931163	-3.100462	-2.188367
H	1.760717	-3.402899	-1.530972
H	-0.011894	-3.500501	-1.784351
H	1.101516	-3.500597	-3.201553
C	-3.120518	0.902190	-1.950070
H	-3.326988	-0.176221	-2.032954
H	-3.557302	1.270406	-1.008906
H	-3.575804	1.434576	-2.801214
C	-0.445949	-0.934586	-3.551472
H	-1.344591	-1.520858	-3.291001
H	-0.067726	-1.308948	-4.518817

C	-0.768445	0.565460	-3.617603
H	0.122773	1.144646	-3.918354
H	-1.564962	0.779795	-4.351535
C	-1.834770	2.895977	4.232407
H	-0.915343	3.498076	4.156309
H	-2.392211	2.984376	3.286987
H	-2.450610	3.271140	5.066137
C	-0.625062	1.175723	6.199844
H	-0.426131	0.152196	6.553707
H	0.329823	1.722065	6.155450
H	-1.304457	1.682481	6.905433
C	-3.161674	-2.241211	2.098981
H	-3.720479	-1.328517	1.841722
H	-2.695650	-2.629814	1.179422
H	-3.848625	-2.997128	2.514230
C	-1.225496	-3.497420	3.828043
H	-0.716435	-3.965701	2.970993
H	-0.502467	-3.387279	4.650686
H	-2.064254	-4.135361	4.151468
C	-2.794101	-1.251966	4.827940
H	-2.205758	-1.539212	5.717334
H	-3.753521	-1.795370	4.879784
C	-3.006132	0.269925	4.784697
H	-3.664482	0.552250	3.943791
H	-3.472423	0.640227	5.714557

3

E=-3199.732500 Hartrees/particle

Mo	-1.270893	-1.137449	0.106401
Mo	-0.616093	1.623464	0.008649
Mo	1.423592	-0.306426	0.100332
S	0.472331	-1.981369	-1.244839
S	-2.202695	0.509636	-1.349591
S	-0.167655	0.121211	1.839609
S	1.400360	1.657482	-1.223188
P	-2.659779	-2.816365	-1.215730
P	-0.874569	-3.353786	1.490383
P	3.580530	-0.967866	-1.038708
P	3.156783	0.825401	1.618540
P	-1.030801	3.797151	-1.212043
P	-2.492426	2.651404	1.424803
H	-0.110063	2.870184	1.123015
H	2.158406	-1.355172	1.283152
C	0.307032	5.065510	-1.067767
H	0.481848	5.294564	-0.005229
H	1.237916	4.660656	-1.494286
H	0.022626	5.985576	-1.604228
C	-4.233188	2.090686	1.148494
H	-4.524326	2.288983	0.105220
H	-4.276464	1.005444	1.317568

H	-4.922639	2.613632	1.831523
C	-2.316626	2.630853	3.261398
H	-2.383736	1.592755	3.622074
H	-1.329081	3.035211	3.533159
H	-3.108749	3.234970	3.733608
C	-1.361220	3.750532	-3.030060
H	-0.479494	3.339341	-3.546677
H	-2.222682	3.093776	-3.227788
H	-1.569596	4.762933	-3.413853
C	-2.587693	4.470829	1.013043
H	-1.737746	4.967258	1.513192
H	-3.517081	4.891025	1.435690
C	-2.524390	4.669222	-0.509378
H	-3.413659	4.232340	-0.997791
H	-2.491319	5.739106	-0.779461
C	4.113070	-2.724208	-0.816718
H	4.220257	-2.945599	0.256359
H	3.339991	-3.388795	-1.233145
H	5.070699	-2.906046	-1.331622
C	3.722400	-0.717573	-2.863810
H	2.982811	-1.354566	-3.374485
H	3.505308	0.334906	-3.104119
H	4.734311	-0.978975	-3.214892
C	3.036277	0.526688	3.435467
H	2.122409	1.003100	3.823502
H	2.977532	-0.556971	3.620632
H	3.914844	0.946155	3.952664
C	3.365365	2.662647	1.515681
H	3.610881	2.949903	0.481640
H	2.414719	3.146608	1.789581
H	4.165436	2.996062	2.196974
C	4.849965	0.166829	1.182524
H	4.953624	-0.811743	1.683572
H	5.619987	0.837287	1.602156
C	5.000389	0.021704	-0.339928
H	4.977259	1.009684	-0.833851
H	5.957064	-0.459765	-0.608254
C	0.777177	-4.175410	1.340255
H	0.975118	-4.440355	0.290612
H	1.562271	-3.479951	1.674232
H	0.789528	-5.087752	1.959620
C	-1.124004	-3.355534	3.321504
H	-0.407456	-2.654071	3.777961
H	-2.150335	-3.035522	3.547516
H	-0.951895	-4.366744	3.726394
C	-2.038347	-4.663276	0.835635
H	-2.998559	-4.484971	1.344528
H	-1.658811	-5.660255	1.121246
C	-4.500233	-2.744071	-1.049927
H	-4.788785	-2.938872	-0.006739

H	-4.847835	-1.738938	-1.337870
H	-4.964207	-3.494300	-1.711498
C	-2.202862	-4.552530	-0.690679
H	-1.257253	-4.794002	-1.208961
H	-2.969115	-5.257532	-1.059219
C	-2.435424	-2.846114	-3.049540
H	-2.790058	-1.890745	-3.468336
H	-1.368207	-2.963666	-3.292619
H	-3.011520	-3.674871	-3.493016
O	-2.947149	-0.986511	1.435378
C	-3.926783	-1.561631	2.072647
H	-4.651654	-0.812574	2.478689
O	-4.116642	-2.774460	2.273423

H_2

E=-1.176599 Hartrees/particle

H	0.000000	0.000000	0.024745
H	0.000000	0.000000	0.775255

TS2

E=-3199.693529 Hartrees/particle

Mo	0.202026	1.686325	-0.015436
Mo	1.501506	-0.795319	0.162022
Mo	-1.273444	-0.772588	-0.279969
S	-1.363657	1.185404	-1.696683
S	2.309148	1.143614	-0.915692
S	-0.140291	0.026776	1.695704
S	0.344050	-1.945458	-1.523084
P	0.757410	3.949069	-1.015106
P	-1.447610	3.259257	1.216127
P	-2.935851	-1.810793	-1.895530
P	-1.596176	-3.183409	0.806107
P	3.510594	-2.031018	-0.756493
P	3.200053	-0.468665	2.047683
H	0.897480	2.644365	1.269343
H	1.610753	-2.132558	1.283004
H	-2.672910	-1.199632	1.016788
C	-3.436138	-0.312397	1.395610
O	-3.910903	-0.563538	2.507770
O	-3.530572	0.590108	0.531216
C	-0.676915	4.958505	1.173740
H	0.215001	4.942068	1.823519
H	-1.387655	5.694340	1.588626
C	-0.301722	5.302610	-0.276442
H	-1.206685	5.396122	-0.902401
H	0.240944	6.261382	-0.341433
C	-3.132801	3.627148	0.536940
H	-3.063228	3.818541	-0.545546
H	-3.776696	2.750171	0.688990
H	-3.552778	4.511283	1.044869

C	-1.766628	2.961312	3.009454
H	-2.327173	2.020458	3.125436
H	-0.806127	2.868911	3.539801
H	-2.350973	3.790803	3.440896
C	2.470820	4.576660	-0.709989
H	2.652823	4.625801	0.374904
H	3.204620	3.888925	-1.156926
H	2.587123	5.580123	-1.151270
C	0.556627	4.172578	-2.839087
H	1.234996	3.480636	-3.363087
H	-0.479380	3.930838	-3.122978
H	0.791668	5.208888	-3.132883
C	4.004781	1.187355	2.247219
H	4.547249	1.452210	1.326468
H	3.228566	1.948153	2.423628
H	4.706410	1.167564	3.097484
C	2.651690	-0.867458	3.763525
H	1.880385	-0.146574	4.076636
H	2.217963	-1.878920	3.779762
H	3.504671	-0.815133	4.459833
C	4.640594	-1.620982	1.765490
H	5.491646	-1.301755	2.391651
H	4.329006	-2.621740	2.113016
C	5.017406	-1.649952	0.275822
H	5.805450	-2.395408	0.071135
H	5.397228	-0.665923	-0.051943
C	4.046694	-1.680228	-2.488646
H	3.247375	-1.987407	-3.181666
H	4.218937	-0.599252	-2.607178
H	4.969761	-2.233146	-2.728055
C	3.420178	-3.877543	-0.718681
H	3.245811	-4.218946	0.313362
H	2.578048	-4.212325	-1.344337
H	4.357503	-4.315288	-1.099725
C	-4.668736	-1.164000	-1.894553
H	-5.120454	-1.262469	-0.896986
H	-4.654624	-0.097197	-2.164939
H	-5.269650	-1.724708	-2.629360
C	-2.516089	-1.796302	-3.693934
H	-2.498040	-0.753545	-4.048703
H	-1.519673	-2.238725	-3.845062
H	-3.267143	-2.365061	-4.266370
C	-3.136901	-3.630340	-1.504582
H	-2.291911	-4.153373	-1.987145
H	-4.064363	-4.002815	-1.973555
C	-0.349417	-4.519798	0.526456
H	-0.216257	-4.701703	-0.550456
H	0.615910	-4.202394	0.948744
H	-0.693720	-5.443999	1.018949
C	-3.138619	-3.886935	0.012619

H	-4.013795	-3.416610	0.495253
H	-3.192184	-4.968242	0.230018
C	-1.922571	-3.300943	2.621280
H	-0.992857	-3.060819	3.161377
H	-2.698235	-2.571872	2.906467
H	-2.246932	-4.319925	2.889303

CO_2
E=-188.588684 Hartrees/particle

O	0.000000	0.000000	1.181403
C	0.000000	0.000000	0.000000
O	0.000000	0.000000	-1.181403

4
E=-3390.691473 Hartrees/particle

Mo	-1.123066	-1.135921	-0.039228
Mo	-0.522840	1.611675	-0.068826
Mo	1.551464	-0.305814	0.001771
S	0.642689	-1.998164	-1.364142
S	-2.134197	0.497688	-1.411627
S	-0.067204	0.085127	1.735716
S	1.493114	1.685632	-1.295515
P	-2.680220	-2.749168	-1.201718
P	-0.845702	-3.243403	1.437253
P	3.707182	-0.992413	-1.127143
P	3.307151	0.754221	1.554575
P	-1.019891	3.762555	-1.308547
P	-2.503039	2.559854	1.313475
H	-2.451374	-1.331821	1.106737
H	2.259230	-1.392443	1.171554
C	0.243461	5.099146	-1.135878
H	0.338931	5.374051	-0.072914
H	1.214226	4.724754	-1.497443
H	-0.054239	5.983250	-1.723810
C	-4.214245	1.921440	1.002435
H	-4.445957	1.959510	-0.072788
H	-4.281466	0.875976	1.336657
H	-4.945022	2.528056	1.562877
C	-2.416651	2.544254	3.158498
H	-2.512197	1.504470	3.508193
H	-1.451687	2.946555	3.500672
H	-3.238032	3.145177	3.582674
C	-1.299375	3.674736	-3.132957
H	-0.376389	3.324575	-3.621712
H	-2.105617	2.955810	-3.346671
H	-1.569574	4.666539	-3.531731
C	-2.661888	4.367692	0.873765
H	-1.838368	4.905825	1.376445
H	-3.615580	4.754637	1.274115
C	-2.579659	4.550112	-0.650688

H	-3.431928	4.059944	-1.154188
H	-2.599841	5.617901	-0.930612
C	4.133482	-2.789473	-1.029090
H	4.213565	-3.090356	0.027088
H	3.330270	-3.378602	-1.498763
H	5.087788	-2.986397	-1.544540
C	3.911265	-0.614607	-2.924449
H	3.147166	-1.165721	-3.495534
H	3.762493	0.464090	-3.088925
H	4.913814	-0.910753	-3.274835
C	3.109661	0.553338	3.378387
H	2.222096	1.112863	3.712068
H	2.963629	-0.512779	3.611064
H	3.999969	0.931303	3.907495
C	3.727955	2.549961	1.372774
H	3.889032	2.790125	0.310370
H	2.895116	3.162328	1.747256
H	4.636597	2.785198	1.951596
C	4.945303	-0.069074	1.198983
H	4.913425	-1.073763	1.656190
H	5.753199	0.498498	1.692834
C	5.170836	-0.161903	-0.318171
H	5.265435	0.844930	-0.762491
H	6.093363	-0.718047	-0.559910
C	0.656211	-4.299493	1.201975
H	0.743534	-4.602510	0.147626
H	1.548841	-3.709556	1.462625
H	0.591138	-5.192367	1.845553
C	-0.904208	-3.068732	3.274911
H	0.004194	-2.543950	3.610634
H	-1.781287	-2.476275	3.574064
H	-0.947297	-4.061635	3.752132
C	-2.255723	-4.399327	1.038344
H	-3.152992	-4.010849	1.552812
H	-2.033900	-5.399053	1.451467
C	-4.483106	-2.394401	-1.014803
H	-4.747501	-2.414537	0.054686
H	-4.693060	-1.390136	-1.415690
H	-5.081592	-3.141808	-1.561821
C	-2.485227	-4.464265	-0.481774
H	-1.623720	-4.932182	-0.990811
H	-3.377612	-5.066836	-0.726626
C	-2.487999	-3.014141	-3.020428
H	-2.702277	-2.069944	-3.545897
H	-1.451387	-3.313399	-3.240489
H	-3.182945	-3.793856	-3.373769
H	-3.280620	-1.115761	2.288966
O	-3.802559	-0.931890	3.140824
C	-4.822813	-1.804785	3.241055
H	-5.404247	-1.603620	4.163602

O	-5.085471	-2.698042	2.444222
H	-0.041433	2.856894	1.083990
O	0.954527	3.866452	3.111900
H	0.556855	3.495906	2.253943
C	0.786515	5.201818	3.148528
H	1.228026	5.613509	4.078663
O	0.229585	5.880342	2.293524

5

E=-3580.470947 Hartrees/particle

Mo	-1.135749	-1.196417	-0.078057
Mo	-0.502783	1.541272	-0.054818
Mo	1.559988	-0.379875	-0.031476
S	0.634426	-2.061751	-1.400076
S	-2.120947	0.473431	-1.415990
S	-0.052545	-0.024578	1.719450
S	1.479485	1.596302	-1.332258
P	-2.780568	-2.694287	-1.284307
P	-0.978075	-3.382677	1.315335
P	3.705980	-1.037833	-1.201907
P	3.347278	0.796380	1.439295
P	-0.971405	3.729436	-1.241517
P	-2.474188	2.484099	1.344822
H	-2.458403	-1.380722	1.072303
C	0.319097	5.033417	-1.026627
H	0.424846	5.265939	0.045516
H	1.280878	4.654402	-1.406702
H	0.036605	5.944551	-1.579677
C	-4.188302	1.871676	1.001589
H	-4.424098	1.982281	-0.067581
H	-4.256925	0.805242	1.260619
H	-4.915592	2.441024	1.603923
C	-2.397607	2.414469	3.189104
H	-2.518453	1.368248	3.510322
H	-1.426663	2.786595	3.547944
H	-3.208700	3.021486	3.624366
C	-1.251004	3.696625	-3.067306
H	-0.339179	3.330579	-3.565221
H	-2.079515	3.009420	-3.299676
H	-1.490150	4.706336	-3.439852
C	-2.606583	4.304501	0.955593
H	-1.776985	4.814763	1.476826
H	-3.555678	4.694080	1.364082
C	-2.516233	4.528307	-0.562586
H	-3.376890	4.070880	-1.082327
H	-2.513645	5.603798	-0.811926
C	4.173006	-2.819383	-1.061933
H	4.314188	-3.073523	0.001142
H	3.361161	-3.439360	-1.473758
H	5.104250	-3.012962	-1.619626

C	3.843625	-0.698097	-3.012521
H	3.075543	-1.279774	-3.546682
H	3.666649	0.372742	-3.198391
H	4.841802	-0.980664	-3.385942
C	3.205509	0.722293	3.278950
H	2.345678	1.334849	3.592103
H	3.041891	-0.314525	3.607749
H	4.121828	1.118111	3.747335
C	3.708693	2.588432	1.141392
H	3.931460	2.755921	0.076830
H	2.832645	3.195066	1.411688
H	4.570607	2.899422	1.754851
C	4.990808	-0.002418	1.060854
H	5.004745	-0.992551	1.550657
H	5.798479	0.607075	1.503052
C	5.169094	-0.144815	-0.460043
H	5.231570	0.845116	-0.946108
H	6.095717	-0.691618	-0.707568
C	0.387373	-4.576259	0.939227
H	0.366536	-4.848587	-0.127304
H	1.358026	-4.107564	1.155480
H	0.270707	-5.482342	1.556612
C	-0.938465	-3.293274	3.159212
H	0.040430	-2.901867	3.476719
H	-1.726816	-2.617835	3.522693
H	-1.083743	-4.297402	3.590785
C	-2.501986	-4.386946	0.931086
H	-3.355743	-3.908216	1.443725
H	-2.381209	-5.403604	1.344858
C	-4.554139	-2.209147	-1.107661
H	-4.827779	-2.218861	-0.040177
H	-4.688059	-1.189867	-1.502712
H	-5.198848	-2.909317	-1.664544
C	-2.732189	-4.427115	-0.588507
H	-1.911406	-4.961370	-1.099140
H	-3.672036	-4.948161	-0.841329
C	-2.581427	-2.931756	-3.105386
H	-2.713897	-1.961058	-3.609386
H	-1.567870	-3.304953	-3.319628
H	-3.329034	-3.646376	-3.487364
H	-3.230892	-1.161786	2.298316
O	-3.710962	-0.991563	3.176089
C	-4.814737	-1.760291	3.239042
H	-5.352905	-1.573875	4.190329
O	-5.184807	-2.555586	2.383330
H	-0.008756	2.760156	1.120260
O	0.977644	3.639638	3.215939
H	0.583568	3.313002	2.339245
C	0.849171	4.977854	3.294525
H	1.286089	5.345416	4.245000

O	0.327786	5.699554	2.452590
H	2.319602	-1.478572	1.123441
O	2.639365	-2.835757	3.168497
H	2.551439	-2.305050	2.307453
C	3.862688	-3.394125	3.231473
H	3.963284	-3.982810	4.165609
O	4.748528	-3.278955	2.392458

6

E=-3389.512806 Hartrees/particle

Mo	-1.209218	-1.077202	0.136394
Mo	-0.554331	1.670745	-0.029760
Mo	1.491775	-0.273486	0.051052
S	0.493221	-1.955968	-1.238881
S	-2.128610	0.534843	-1.368043
S	-0.059093	0.206409	1.820751
S	1.453431	1.689137	-1.275756
P	-2.648297	-2.769727	-1.115759
P	-0.813401	-3.261684	1.563331
P	3.585101	-1.037359	-1.152759
P	3.340692	0.772979	1.502653
P	-0.997458	3.820252	-1.294428
P	-2.543523	2.659125	1.296876
H	2.215836	-1.334287	1.228923
C	0.323232	5.104390	-1.160412
H	0.454621	5.377168	-0.100913
H	1.269861	4.694004	-1.545419
H	0.042994	5.997470	-1.743240
C	-4.251939	2.096742	0.863367
H	-4.475743	2.367092	-0.180127
H	-4.300146	1.002678	0.955266
H	-4.988517	2.570728	1.533280
C	-2.525211	2.583448	3.141186
H	-2.631977	1.532470	3.451465
H	-1.567994	2.967843	3.525065
H	-3.355863	3.175341	3.559577
C	-1.314824	3.719741	-3.111985
H	-0.417061	3.324421	-3.613190
H	-2.154410	3.032564	-3.299997
H	-1.551096	4.716375	-3.520130
C	-2.618246	4.481158	0.899564
H	-1.780479	4.974837	1.423060
H	-3.561350	4.900633	1.292077
C	-2.509265	4.686073	-0.620877
H	-3.385815	4.259649	-1.139998
H	-2.463015	5.757852	-0.881436
C	3.980484	-2.838335	-1.019112
H	4.098504	-3.111502	0.040923
H	3.148839	-3.424858	-1.440010
H	4.908930	-3.068154	-1.567030

C	3.703026	-0.714634	-2.967789
H	2.896485	-1.259813	-3.483272
H	3.573428	0.362907	-3.154263
H	4.678295	-1.046737	-3.360060
C	3.208376	0.585420	3.332762
H	2.339332	1.157010	3.693881
H	3.060990	-0.477539	3.578233
H	4.121957	0.958844	3.823990
C	3.764786	2.563940	1.294063
H	3.914235	2.792603	0.227661
H	2.936739	3.181116	1.671543
H	4.680885	2.802623	1.859452
C	4.950857	-0.073600	1.082929
H	4.936727	-1.065534	1.567877
H	5.786792	0.500082	1.519567
C	5.097353	-0.208303	-0.440701
H	5.181905	0.784256	-0.917584
H	5.998279	-0.784984	-0.713491
C	0.817345	-4.117444	1.384131
H	0.976602	-4.414266	0.336509
H	1.625279	-3.428593	1.674175
H	0.832827	-5.013043	2.027247
C	-1.011012	-3.205899	3.399350
H	-0.270190	-2.505581	3.816921
H	-2.024940	-2.860362	3.643228
H	-0.846310	-4.208518	3.827851
C	-2.020242	-4.563049	0.976879
H	-2.966471	-4.345542	1.497114
H	-1.659066	-5.558863	1.288860
C	-4.483259	-2.666397	-0.911696
H	-4.752547	-2.844145	0.139768
H	-4.824529	-1.660916	-1.205241
H	-4.970820	-3.419416	-1.552848
C	-2.208697	-4.496717	-0.549102
H	-1.276007	-4.768700	-1.075616
H	-2.992117	-5.199838	-0.883316
C	-2.458535	-2.855207	-2.951470
H	-2.814785	-1.911414	-3.394137
H	-1.396532	-2.987205	-3.209465
H	-3.047015	-3.693599	-3.359082
O	-2.853245	-0.871021	1.486190
C	-3.831887	-1.400681	2.165563
H	-4.538539	-0.622074	2.545435
O	-4.032683	-2.599958	2.425260
H	-0.076156	2.969705	1.060339
O	0.970701	3.839179	3.129948
H	0.556902	3.495295	2.268938
C	0.872534	5.181460	3.166351
H	1.331396	5.570211	4.098022
O	0.354949	5.887325	2.308638

7

E=-3579.290803 Hartrees/particle

Mo	-1.233616	-1.144176	0.081941
Mo	-0.553895	1.601015	-0.022345
Mo	1.480777	-0.349084	0.018417
S	0.464469	-1.987096	-1.318715
S	-2.128048	0.507985	-1.382335
S	-0.070887	0.092151	1.799333
S	1.421947	1.596823	-1.314813
P	-2.758337	-2.724012	-1.215898
P	-0.890058	-3.435765	1.371471
P	3.568012	-1.109963	-1.207054
P	3.362530	0.810114	1.389347
P	-0.962653	3.789335	-1.233674
P	-2.521698	2.593067	1.328586
C	0.386483	5.038990	-1.063835
H	0.529408	5.272789	0.003512
H	1.322223	4.622447	-1.468201
H	0.123039	5.956947	-1.614874
C	-4.236986	2.064983	0.881304
H	-4.455065	2.361780	-0.156241
H	-4.303232	0.969925	0.949369
H	-4.967546	2.535035	1.560380
C	-2.500414	2.466084	3.169739
H	-2.614482	1.408265	3.452667
H	-1.539492	2.832603	3.561427
H	-3.325496	3.053223	3.605361
C	-1.282640	3.744768	-3.052653
H	-0.398457	3.332902	-3.564527
H	-2.145078	3.091740	-3.258169
H	-1.485278	4.759156	-3.434145
C	-2.566341	4.424829	0.980406
H	-1.719602	4.889279	1.516036
H	-3.501584	4.849443	1.385771
C	-2.455542	4.667421	-0.534327
H	-3.341169	4.272847	-1.063278
H	-2.387603	5.744472	-0.766510
C	3.970200	-2.904091	-1.039451
H	4.115164	-3.144701	0.026254
H	3.131844	-3.501806	-1.430684
H	4.886471	-3.141756	-1.604756
C	3.653122	-0.812862	-3.027864
H	2.843962	-1.372718	-3.523072
H	3.512868	0.260599	-3.229429
H	4.625321	-1.143553	-3.428887
C	3.284024	0.810726	3.233418
H	2.451833	1.457849	3.551114
H	3.113727	-0.206537	3.614297
H	4.227177	1.205229	3.646411

C	3.770386	2.578133	1.023070
H	3.954742	2.714372	-0.052841
H	2.924976	3.217438	1.314972
H	4.665365	2.875737	1.594326
C	4.960407	-0.064240	0.985982
H	4.948299	-1.039800	1.504101
H	5.804765	0.523287	1.387210
C	5.085516	-0.255216	-0.534577
H	5.168709	0.718034	-1.049851
H	5.981768	-0.844974	-0.794636
C	0.636431	-4.406277	0.969713
H	0.590593	-4.748505	-0.075689
H	1.536096	-3.786092	1.084340
H	0.697011	-5.282967	1.635583
C	-0.950154	-3.491287	3.215469
H	-0.096484	-2.926541	3.620768
H	-1.895021	-3.039601	3.549032
H	-0.893432	-4.535950	3.564706
C	-2.223379	-4.615005	0.804410
H	-3.130621	-4.335143	1.363602
H	-1.935218	-5.643999	1.083572
C	-4.575523	-2.494526	-0.966136
H	-4.841437	-2.723230	0.076455
H	-4.842702	-1.448436	-1.184056
H	-5.131666	-3.165158	-1.641680
C	-2.447515	-4.496465	-0.712781
H	-1.559608	-4.835036	-1.276700
H	-3.298123	-5.120161	-1.039900
C	-2.593661	-2.748651	-3.054463
H	-2.874262	-1.760709	-3.453056
H	-1.547937	-2.955699	-3.329642
H	-3.251449	-3.519235	-3.488614
O	-2.865211	-0.966435	1.430617
C	-3.821052	-1.461414	2.167954
H	-4.534782	-0.669091	2.502887
O	-3.994016	-2.641345	2.516134
H	-0.051539	2.869480	1.093245
O	1.020862	3.637858	3.193388
H	0.594913	3.333666	2.324167
C	0.959119	4.980772	3.271671
H	1.432072	5.328290	4.212442
O	0.456548	5.725133	2.437990
H	2.237995	-1.433195	1.188123
O	2.549548	-2.599456	3.371761
H	2.436520	-2.141877	2.474203
C	3.701615	-3.297373	3.368812
H	3.833098	-3.817579	4.338909
O	4.499801	-3.356813	2.440848

8

E=-3388.335315 Hartrees/particle

Mo	-1.247288	-1.282510	0.085516
Mo	-0.576447	1.460453	0.069656
Mo	1.466961	-0.469146	0.221945
S	0.511595	-2.066717	-1.254217
S	-2.129780	0.399485	-1.345564
S	-0.211074	-0.068901	1.888806
S	1.423033	1.417799	-1.166667
P	-2.786032	-2.814802	-1.285999
P	-1.011202	-3.586790	1.350216
P	3.670442	-0.908137	-1.004449
P	3.161967	0.949614	1.660905
P	-0.897189	3.656892	-1.149133
P	-2.493135	2.522535	1.405508
C	0.466264	4.888533	-0.942380
H	0.606561	5.106802	0.127369
H	1.403182	4.466685	-1.338241
H	0.225040	5.818743	-1.482553
C	-4.224069	1.991788	1.016352
H	-4.470709	2.296342	-0.012894
H	-4.294251	0.895906	1.070318
H	-4.937350	2.454201	1.718233
C	-2.404284	2.464486	3.245338
H	-2.516525	1.419559	3.575641
H	-1.420971	2.839545	3.569359
H	-3.203007	3.083764	3.685943
C	-1.149842	3.620587	-2.978770
H	-0.256351	3.189761	-3.457931
H	-2.017633	2.985584	-3.215639
H	-1.316721	4.638919	-3.366647
C	-2.536091	4.345716	1.011219
H	-1.701969	4.818657	1.558396
H	-3.477006	4.777486	1.394702
C	-2.397451	4.559294	-0.503763
H	-3.274517	4.152128	-1.037604
H	-2.323949	5.630640	-0.758995
C	4.503491	-2.538533	-0.750310
H	4.743158	-2.670104	0.314999
H	3.828290	-3.348286	-1.069935
H	5.428578	-2.581003	-1.348923
C	3.665250	-0.747460	-2.845177
H	3.022560	-1.533468	-3.272564
H	3.260942	0.234764	-3.134183
H	4.688057	-0.858267	-3.241372
C	3.209490	0.751775	3.496144
H	2.227532	1.033637	3.908779
H	3.428940	-0.296474	3.741139
H	3.986104	1.406117	3.925789

C	3.059072	2.789338	1.485063
H	3.188659	3.078086	0.431081
H	2.066443	3.132849	1.815466
H	3.845577	3.259294	2.098724
C	4.893838	0.565230	1.072712
H	5.187060	-0.355974	1.601406
H	5.570753	1.382235	1.378176
C	4.933915	0.354746	-0.451842
H	4.696206	1.290639	-0.989121
H	5.937827	0.032560	-0.780284
C	0.307060	-4.748906	0.776431
H	0.171831	-4.987112	-0.288998
H	1.285392	-4.264239	0.900700
H	0.266708	-5.678012	1.368926
C	-0.865511	-3.672711	3.187701
H	0.124526	-3.284579	3.474104
H	-1.634753	-3.033062	3.643209
H	-0.966680	-4.714527	3.534602
C	-2.565787	-4.544696	0.954136
H	-3.399714	-4.070503	1.497705
H	-2.459553	-5.576414	1.333560
C	-4.562872	-2.310130	-1.314779
H	-4.922383	-2.199363	-0.281850
H	-4.655531	-1.339618	-1.827244
H	-5.159253	-3.068184	-1.848364
C	-2.825141	-4.544427	-0.562667
H	-2.052615	-5.130247	-1.090950
H	-3.798409	-5.007308	-0.801155
C	-2.408804	-3.094465	-3.071735
H	-2.483535	-2.135834	-3.609769
H	-1.382586	-3.478760	-3.178150
H	-3.121413	-3.813111	-3.508799
O	-3.068769	-1.389121	1.232476
C	-3.443692	-1.191899	2.477745
H	-4.558030	-1.224796	2.567031
O	-2.738915	-0.999161	3.477925
O	2.053694	-2.029213	1.564273
C	2.991660	-2.597925	2.267744
H	2.671189	-3.598395	2.650848
O	4.123966	-2.163211	2.542546
H	-0.090748	2.672994	1.224645

9

E=-3578.113982 Hartrees/particle

Mo	-1.202505	-1.211019	0.116627
Mo	-0.518705	1.520548	0.046935
Mo	1.523720	-0.430732	0.184529
S	0.514867	-2.024805	-1.248243
S	-2.056495	0.445075	-1.358934
S	-0.121275	0.021169	1.885468

S	1.444715	1.425000	-1.241897
P	-2.772398	-2.757031	-1.208702
P	-0.967736	-3.493373	1.416187
P	3.655776	-1.019169	-1.100475
P	3.362600	0.981330	1.469486
P	-0.842571	3.691884	-1.222728
P	-2.534726	2.580405	1.293308
C	0.493492	4.941873	-0.971056
H	0.564126	5.186370	0.101209
H	1.454489	4.521264	-1.306123
H	0.271616	5.854461	-1.548792
C	-4.224057	2.007842	0.799890
H	-4.403057	2.258707	-0.257114
H	-4.287333	0.914711	0.906060
H	-4.986907	2.495723	1.429000
C	-2.595095	2.541852	3.136686
H	-2.692614	1.496873	3.470296
H	-1.666736	2.957216	3.555699
H	-3.455666	3.130323	3.495634
C	-1.024056	3.617525	-3.058848
H	-0.101628	3.203512	-3.496016
H	-1.866164	2.957778	-3.319650
H	-1.200791	4.625600	-3.468587
C	-2.573276	4.394609	0.864074
H	-1.759667	4.884080	1.428415
H	-3.531327	4.827015	1.202040
C	-2.380232	4.579585	-0.649562
H	-3.231415	4.155389	-1.210849
H	-2.306874	5.647286	-0.920049
C	4.344605	-2.710918	-0.818568
H	4.638610	-2.820586	0.235777
H	3.577828	-3.464212	-1.058744
H	5.224909	-2.864176	-1.464147
C	3.605946	-0.894034	-2.941937
H	2.888411	-1.631324	-3.335584
H	3.275010	0.113645	-3.236819
H	4.603998	-1.094760	-3.364705
C	3.446567	0.968116	3.313555
H	2.532991	1.429049	3.719564
H	3.530973	-0.072462	3.657010
H	4.324746	1.542940	3.652219
C	3.453287	2.787161	1.071787
H	3.641626	2.927605	-0.003443
H	2.505254	3.282670	1.321195
H	4.272474	3.244020	1.651391
C	5.041292	0.388875	0.904679
H	5.235610	-0.536358	1.470952
H	5.800467	1.141817	1.180933
C	5.049387	0.123975	-0.610183
H	4.913994	1.059163	-1.182651

H	6.008473	-0.319699	-0.930990
C	0.318872	-4.678690	0.819153
H	0.150491	-4.928305	-0.239021
H	1.306679	-4.205164	0.908616
H	0.285144	-5.600082	1.423850
C	-0.771388	-3.550878	3.249306
H	0.225439	-3.158940	3.504524
H	-1.529154	-2.903346	3.712985
H	-0.863633	-4.587402	3.613823
C	-2.548140	-4.428595	1.079529
H	-3.359627	-3.920705	1.626495
H	-2.456736	-5.451813	1.484764
C	-4.539090	-2.220580	-1.250593
H	-4.895571	-2.073751	-0.221096
H	-4.616188	-1.264542	-1.791965
H	-5.149189	-2.983591	-1.761078
C	-2.839414	-4.463348	-0.430882
H	-2.093959	-5.084765	-0.957085
H	-3.829277	-4.906468	-0.635741
C	-2.401282	-3.101811	-2.983862
H	-2.446785	-2.159422	-3.552670
H	-1.387072	-3.520294	-3.075637
H	-3.134481	-3.812867	-3.398539
O	-3.010575	-1.265996	1.275231
C	-3.378175	-1.064148	2.522508
H	-4.492523	-1.041765	2.610091
O	-2.664769	-0.917959	3.523894
H	-0.017728	2.782198	1.175062
O	0.793870	3.557454	3.409828
H	0.462613	3.242162	2.505321
C	0.770727	4.903991	3.442324
H	1.145808	5.266492	4.420647
O	0.391547	5.635650	2.535405
O	2.130208	-1.955772	1.540301
C	3.025973	-2.535682	2.290143
H	2.677978	-3.535712	2.649028
O	4.144579	-2.111275	2.625023

10

E=-3576.937857 Hartrees/particle

Mo	-1.179708	-1.175292	0.176698
Mo	-0.519333	1.570295	0.211975
Mo	1.543330	-0.382337	0.273507
S	0.539008	-1.945584	-1.194265
S	-1.993641	0.511585	-1.259785
S	-0.110824	-0.015079	1.992406
S	1.416234	1.455422	-1.151652
P	-2.742664	-2.659353	-1.230281
P	-0.976245	-3.504712	1.382772
P	3.662334	-0.982589	-1.036638

P	3.421867	1.030074	1.530539
P	-0.928698	3.682809	-1.188824
P	-2.682248	2.577247	1.341070
C	0.363143	4.984998	-0.982358
H	0.454638	5.239988	0.082969
H	1.330210	4.591174	-1.332279
H	0.092883	5.879460	-1.566741
C	-4.318842	1.940219	0.763002
H	-4.435765	2.143928	-0.312520
H	-4.369015	0.851557	0.912026
H	-5.130998	2.435783	1.319766
C	-2.866258	2.622415	3.179537
H	-2.837756	1.594509	3.573409
H	-2.050913	3.204788	3.633692
H	-3.827345	3.095167	3.441011
C	-1.088094	3.521486	-3.019279
H	-0.146412	3.124613	-3.430412
H	-1.902855	2.821750	-3.261382
H	-1.297458	4.504325	-3.472450
C	-2.746079	4.375317	0.845190
H	-1.966722	4.900792	1.422997
H	-3.724161	4.795133	1.138890
C	-2.509393	4.528989	-0.666353
H	-3.331514	4.067217	-1.240924
H	-2.463768	5.592308	-0.959028
C	4.302146	-2.696978	-0.778675
H	4.605084	-2.829370	0.270421
H	3.512496	-3.425367	-1.020220
H	5.172542	-2.866376	-1.433523
C	3.606473	-0.828854	-2.874916
H	2.866732	-1.539516	-3.276251
H	3.303288	0.192197	-3.152854
H	4.596805	-1.051362	-3.304649
C	3.533851	1.070869	3.373913
H	2.647527	1.573920	3.788423
H	3.589609	0.037313	3.743854
H	4.437753	1.625001	3.677815
C	3.577999	2.816473	1.067352
H	3.689182	2.911174	-0.023397
H	2.688185	3.380990	1.379589
H	4.469693	3.234260	1.564134
C	5.077566	0.360971	0.983902
H	5.226027	-0.572426	1.551355
H	5.864075	1.079753	1.274199
C	5.092933	0.105386	-0.531562
H	5.003337	1.048276	-1.099818
H	6.035792	-0.375966	-0.845597
C	0.357411	-4.646233	0.808268
H	0.232816	-4.875084	-0.260453
H	1.330133	-4.152856	0.945133

H	0.321662	-5.581452	1.391094
C	-0.847574	-3.599593	3.219788
H	0.135500	-3.203290	3.518895
H	-1.625665	-2.970917	3.675115
H	-0.941552	-4.644809	3.557781
C	-2.526479	-4.452374	0.952761
H	-3.362913	-3.993567	1.505712
H	-2.420634	-5.493722	1.304678
C	-4.514119	-2.139162	-1.217672
H	-4.861342	-2.062956	-0.177701
H	-4.605354	-1.150618	-1.694236
H	-5.122402	-2.872584	-1.771551
C	-2.777069	-4.409020	-0.564910
H	-1.998933	-4.974289	-1.107095
H	-3.746781	-4.869265	-0.822066
C	-2.381113	-2.870714	-3.027650
H	-2.442307	-1.889113	-3.524413
H	-1.363301	-3.269259	-3.158419
H	-3.109805	-3.557897	-3.487945
O	-2.996539	-1.271021	1.322079
C	-3.365855	-1.068660	2.570103
H	-4.479367	-1.095873	2.664200
O	-2.653297	-0.876153	3.563662
O	0.205645	3.103490	1.491268
C	0.485716	3.097880	2.784668
H	0.163569	2.174848	3.325141
O	1.063777	4.022649	3.364166
O	2.177087	-1.926932	1.580678
C	3.032882	-2.500170	2.382435
H	2.694850	-3.521867	2.684408
O	4.106353	-2.047363	2.811032

TS3

E=-3390.643487 Hartrees/particle

Mo	-0.026145	-0.089528	-0.042896
Mo	-0.050121	0.005424	2.761721
Mo	2.516397	-0.043596	1.468693
S	1.610185	1.526410	-0.287945
S	-1.706319	0.843369	1.295595
S	0.863098	-1.809453	1.436678
S	1.546268	1.672099	2.913202
P	-1.379186	1.020116	-1.871096
P	0.792904	-1.384191	-2.157328
P	4.625311	1.351013	1.464759
P	3.553581	-0.640983	3.855556
P	-1.209764	1.364438	4.569992
P	-1.767495	-1.674399	3.673101
H	-1.189865	-1.290247	-0.635551
H	0.520484	-0.942060	4.114136
H	3.019290	-0.367151	-0.239226

H	5.039049	-3.337879	0.774048
C	4.335980	-2.510236	0.567585
O	4.054229	-1.723139	1.528849
O	3.868646	-2.395693	-0.616992
H	3.267614	-1.263391	-0.423181
C	5.905544	0.859429	0.220437
H	6.194511	-0.191744	0.371134
H	5.487665	0.969434	-0.792582
H	6.799378	1.497119	0.319241
C	4.498462	3.175614	1.208304
H	4.100118	3.369994	0.200188
H	3.803960	3.601283	1.949092
H	5.488810	3.649117	1.311262
C	3.075004	0.219852	5.418472
H	3.143161	1.311678	5.304671
H	2.037010	-0.042564	5.665703
H	3.748395	-0.113115	6.225930
C	3.552551	-2.406642	4.409795
H	2.517685	-2.689374	4.660102
H	3.918368	-3.051109	3.599550
H	4.188394	-2.529373	5.302246
C	5.366905	-0.183628	3.727544
H	5.861500	-0.952769	3.112802
H	5.815376	-0.214944	4.736155
C	5.525402	1.213242	3.100858
H	5.095132	1.986349	3.762337
H	6.589774	1.467258	2.952579
C	2.322785	-0.852835	-3.072629
H	2.401482	0.245295	-3.059393
H	3.220399	-1.274740	-2.599964
H	2.273046	-1.210464	-4.114604
C	-1.286220	2.859302	-2.030665
H	-1.694004	3.320699	-1.117125
H	-0.234141	3.164740	-2.139438
H	-1.865920	3.202449	-2.903556
C	0.956586	-3.224219	-2.107530
H	1.820761	-3.486239	-1.478283
H	0.048705	-3.664542	-1.668463
H	1.107872	-3.624083	-3.123918
C	-3.197103	0.689443	-1.857050
H	-3.366301	-0.397062	-1.931825
H	-3.629369	1.053510	-0.911992
H	-3.679847	1.203821	-2.704453
C	-0.517565	-1.103868	-3.459627
H	-1.407960	-1.691281	-3.172055
H	-0.151383	-1.491742	-4.426486
C	-0.856829	0.392069	-3.548999
H	0.022967	0.979452	-3.866864
H	-1.662918	0.582374	-4.279185
C	-1.825777	3.046888	4.117904

H	-0.967553	3.682911	3.848645
H	-2.493650	2.969055	3.246105
H	-2.367067	3.501777	4.963656
C	-0.275157	1.695677	6.132291
H	-0.004440	0.744941	6.616892
H	0.649076	2.244300	5.894228
H	-0.891446	2.294373	6.823159
C	-3.317444	-2.025782	2.719311
H	-3.839498	-1.083304	2.491916
H	-3.064675	-2.521767	1.769416
H	-3.979306	-2.683704	3.307140
C	-1.167530	-3.368134	4.097356
H	-0.834524	-3.878283	3.180000
H	-0.312826	-3.287220	4.786275
H	-1.972833	-3.953460	4.570753
C	-2.428966	-1.031708	5.296955
H	-1.658163	-1.222782	6.064592
H	-3.328647	-1.606349	5.578316
C	-2.730169	0.470581	5.182812
H	-3.540666	0.654460	4.455462
H	-3.047014	0.898820	6.149680
O	-2.308020	-3.454638	-0.353325
H	-1.862185	-2.542198	-0.456248
C	-3.211017	-3.626686	-1.334917
H	-3.710139	-4.611279	-1.224396
O	-3.461035	-2.829414	-2.232303

TS4

E=-3389.473544 Hartrees/particle

Mo	0.208366	1.716281	0.035124
Mo	1.535538	-0.767910	0.184169
Mo	-1.241313	-0.751850	-0.222516
S	-1.339975	1.202586	-1.645512
S	2.316699	1.190907	-0.871230
S	-0.095020	0.043458	1.745909
S	0.359394	-1.904332	-1.494310
P	0.766237	3.976082	-0.982831
P	-1.557290	3.303014	1.138385
P	-2.920878	-1.786623	-1.826369
P	-1.554648	-3.157860	0.859312
P	3.504925	-2.030879	-0.800051
P	3.293727	-0.517936	2.031913
H	0.884825	2.707209	1.325408
H	1.652541	-2.116391	1.289545
H	-2.630952	-1.167725	1.069601
C	-3.406750	-0.292763	1.473431
O	-3.849317	-0.568973	2.592146
O	-3.536625	0.612613	0.620280
C	-0.807398	5.011490	1.089009
H	0.055128	5.040297	1.777071

H	-1.553696	5.744579	1.442732
C	-0.366812	5.321694	-0.351297
H	-1.237272	5.375521	-1.028027
H	0.157502	6.290920	-0.415055
C	-3.179060	3.626016	0.304162
H	-3.018181	3.802012	-0.770613
H	-3.822327	2.744739	0.423134
H	-3.652237	4.513285	0.757278
C	-2.036883	3.060517	2.905337
H	-2.616807	2.128560	2.992294
H	-1.136866	2.980147	3.533655
H	-2.653334	3.906208	3.252531
C	2.437876	4.641815	-0.561140
H	2.509081	4.757209	0.532537
H	3.215824	3.940666	-0.899719
H	2.583371	5.619778	-1.048917
C	0.685744	4.132539	-2.821506
H	1.412506	3.437660	-3.271396
H	-0.323094	3.858716	-3.167646
H	0.920742	5.162900	-3.135174
C	4.242054	1.065458	2.184174
H	4.719538	1.311079	1.223585
H	3.557041	1.881945	2.454333
H	5.013363	0.966151	2.965920
C	2.755381	-0.851394	3.763955
H	2.039834	-0.075627	4.077678
H	2.257501	-1.832196	3.805318
H	3.624143	-0.843924	4.442396
C	4.644028	-1.768832	1.732365
H	5.509267	-1.527110	2.373709
H	4.258088	-2.753690	2.048756
C	5.029200	-1.777224	0.245368
H	5.767456	-2.566110	0.019398
H	5.473957	-0.811104	-0.052253
C	4.048537	-1.600051	-2.511351
H	3.232601	-1.824507	-3.216565
H	4.273167	-0.523398	-2.563828
H	4.942941	-2.180664	-2.790642
C	3.338235	-3.870893	-0.866712
H	3.171726	-4.264874	0.147790
H	2.472043	-4.137290	-1.492005
H	4.250547	-4.320882	-1.291617
C	-4.648767	-1.127896	-1.818735
H	-5.096156	-1.217862	-0.818572
H	-4.628543	-0.062742	-2.095256
H	-5.256664	-1.688507	-2.547850
C	-2.510830	-1.781321	-3.626928
H	-2.489448	-0.740425	-3.986825
H	-1.518259	-2.230486	-3.783105
H	-3.269028	-2.348279	-4.191654

C	-3.128480	-3.603352	-1.426558
H	-2.293987	-4.133623	-1.919302
H	-4.064716	-3.970323	-1.882071
C	-0.314509	-4.494342	0.555045
H	-0.195801	-4.671137	-0.524386
H	0.656640	-4.181572	0.967160
H	-0.654477	-5.420341	1.047209
C	-3.111300	-3.856172	0.090954
H	-3.977699	-3.381386	0.584693
H	-3.165921	-4.936757	0.311495
C	-1.845717	-3.272362	2.679664
H	-0.906049	-3.031449	3.201910
H	-2.616421	-2.543276	2.977628
H	-2.164543	-4.291315	2.954082
O	1.570782	2.909674	3.707283
H	1.307039	2.815141	2.731750
C	2.045437	4.152337	3.918428
H	2.344068	4.269211	4.979985
O	2.140593	5.036239	3.075053

TS5

E=-3580.419744 Hartrees/particle

Mo	-0.009119	-0.016509	-0.061517
Mo	-0.149060	-0.074389	2.737217
Mo	2.478571	-0.096956	1.520494
S	1.673268	1.557142	-0.186069
S	-1.644759	0.992206	1.265138
S	0.779723	-1.828131	1.356092
S	1.492664	1.520003	3.056107
P	-1.284931	1.176509	-1.896461
P	0.835727	-1.259762	-2.195729
P	4.557444	1.363641	1.532805
P	3.620989	-0.738913	3.897075
P	-1.313364	1.209712	4.604336
P	-2.174057	-1.614702	3.314148
H	-1.198270	-1.156339	-0.721800
H	0.249527	-1.166962	4.084599
H	3.058206	-0.334388	-0.197521
H	4.949571	-3.419686	0.783573
C	4.302852	-2.551666	0.554084
O	3.910123	-1.848347	1.545165
O	4.009066	-2.320597	-0.664542
H	3.358722	-1.166732	-0.419281
C	5.812430	0.955859	0.233640
H	6.153884	-0.084752	0.346685
H	5.353792	1.070077	-0.761041
H	6.679438	1.631635	0.316716
C	4.362111	3.190394	1.346655
H	3.953231	3.414667	0.349396
H	3.658848	3.561337	2.108422

H	5.337643	3.689873	1.466631
C	3.154287	0.109330	5.473869
H	3.280725	1.198633	5.384006
H	2.100380	-0.093035	5.711154
H	3.796513	-0.268865	6.286701
C	3.809814	-2.495766	4.455326
H	2.854538	-2.875109	4.846315
H	4.131765	-3.113883	3.606835
H	4.567915	-2.540682	5.255535
C	5.407212	-0.208393	3.705709
H	5.891568	-0.941536	3.038945
H	5.901381	-0.274796	4.691362
C	5.510450	1.211699	3.131715
H	5.080841	1.949843	3.832123
H	6.561702	1.500953	2.957637
C	2.383164	-0.704306	-3.062424
H	2.451513	0.393941	-3.033856
H	3.271490	-1.125749	-2.572704
H	2.361599	-1.048113	-4.110047
C	-1.134952	3.014156	-2.022181
H	-1.549016	3.473601	-1.110555
H	-0.072934	3.291715	-2.106280
H	-1.686695	3.387048	-2.900999
C	1.005797	-3.098834	-2.176685
H	1.862251	-3.367498	-1.539172
H	0.094081	-3.552288	-1.759790
H	1.175494	-3.479447	-3.197450
C	-3.111506	0.898590	-1.916849
H	-3.308776	-0.180235	-2.025737
H	-3.550200	1.249798	-0.969994
H	-3.565142	1.448878	-2.757722
C	-0.445492	-0.940429	-3.515159
H	-1.350597	-1.518504	-3.256591
H	-0.066185	-1.313284	-4.482886
C	-0.757381	0.563083	-3.579281
H	0.134430	1.140541	-3.881351
H	-1.554562	0.780524	-4.311564
C	-1.753726	2.974056	4.278531
H	-0.828496	3.547661	4.109699
H	-2.378191	3.039042	3.374139
H	-2.296760	3.400004	5.138153
C	-0.427501	1.303312	6.222298
H	-0.264727	0.285195	6.611682
H	0.544957	1.797463	6.072254
H	-1.024876	1.884628	6.944428
C	-3.703146	-1.516791	2.271999
H	-4.052250	-0.475780	2.196907
H	-3.480597	-1.884441	1.259490
H	-4.492482	-2.143307	2.720306
C	-1.916603	-3.441360	3.415515

H	-1.651380	-3.812307	2.413128
H	-1.097321	-3.679871	4.108853
H	-2.842338	-3.935925	3.753365
C	-2.815364	-1.119191	4.993583
H	-2.104523	-1.484048	5.755777
H	-3.787651	-1.611176	5.171551
C	-2.941026	0.412575	5.060265
H	-3.702579	0.776920	4.348363
H	-3.242221	0.750317	6.067100
O	-2.378725	-3.306202	-0.546758
H	-1.902225	-2.407061	-0.610718
C	-3.234699	-3.433770	-1.576314
H	-3.765236	-4.405794	-1.511749
O	-3.417206	-2.611533	-2.467458
O	0.703568	-3.073286	5.622805
H	0.484064	-2.238541	5.086368
C	0.289797	-2.934227	6.896206
H	0.554037	-3.838351	7.480848
O	-0.297623	-1.963319	7.358334

TS6

E=-3579.252196 Hartrees/particle

Mo	0.202946	1.637264	0.096001
Mo	1.558075	-0.825159	0.239154
Mo	-1.238071	-0.836759	-0.176292
S	-1.347838	1.124399	-1.575914
S	2.261493	1.097416	-0.906478
S	-0.085462	-0.046039	1.803310
S	0.381999	-1.981242	-1.427821
P	0.762328	3.903656	-0.908402
P	-1.554885	3.222554	1.210505
P	-2.906948	-1.759407	-1.863338
P	-1.654508	-3.289473	0.784001
P	3.527312	-2.090086	-0.757247
P	3.440043	-0.338624	1.951296
H	0.889894	2.618271	1.390951
H	1.808152	-2.137810	1.397399
H	-2.618531	-1.266067	1.116732
C	-3.387063	-0.388429	1.523742
O	-3.837121	-0.666284	2.638478
O	-3.504479	0.523903	0.674749
C	-0.799301	4.928465	1.181072
H	0.061889	4.945952	1.871008
H	-1.542936	5.660812	1.541590
C	-0.355335	5.251966	-0.255007
H	-1.225699	5.325140	-0.930058
H	0.179909	6.215858	-0.306373
C	-3.170892	3.550968	0.368507
H	-3.002262	3.746368	-0.701716
H	-3.809571	2.663823	0.467844

H	-3.653529	4.427369	0.832594
C	-2.043177	2.961507	2.972027
H	-2.619923	2.026690	3.047070
H	-1.146405	2.879313	3.604704
H	-2.664950	3.801857	3.322629
C	2.443641	4.547682	-0.492087
H	2.529118	4.639746	0.602748
H	3.212428	3.848375	-0.854652
H	2.591578	5.534221	-0.961566
C	0.668333	4.081808	-2.744276
H	1.389532	3.391073	-3.209246
H	-0.344214	3.816423	-3.086151
H	0.904325	5.115522	-3.045978
C	4.363324	1.258786	1.801435
H	4.806143	1.348812	0.797746
H	3.672186	2.101568	1.947898
H	5.158762	1.297456	2.563772
C	3.056534	-0.409040	3.754876
H	2.419730	0.450356	4.016594
H	2.522832	-1.339260	3.997754
H	3.992145	-0.357512	4.336161
C	4.776061	-1.612817	1.701327
H	5.671073	-1.315273	2.275367
H	4.407273	-2.568687	2.114577
C	5.091279	-1.748063	0.203359
H	5.816555	-2.558429	0.014244
H	5.528426	-0.815422	-0.194586
C	3.982447	-1.745061	-2.512956
H	3.143559	-2.034034	-3.166044
H	4.172756	-0.667997	-2.639640
H	4.880461	-2.317334	-2.797947
C	3.387169	-3.930201	-0.699160
H	3.264254	-4.252859	0.347464
H	2.507436	-4.248303	-1.280040
H	4.293684	-4.390379	-1.126144
C	-4.608430	-1.037716	-1.855623
H	-5.086116	-1.177886	-0.875463
H	-4.538145	0.042147	-2.056281
H	-5.215388	-1.523522	-2.637156
C	-2.448395	-1.669454	-3.649536
H	-2.379474	-0.612218	-3.950352
H	-1.469857	-2.147982	-3.807123
H	-3.212475	-2.175537	-4.262167
C	-3.180256	-3.583145	-1.567601
H	-2.350867	-4.116187	-2.065843
H	-4.116298	-3.898371	-2.060378
C	-0.444792	-4.639822	0.413958
H	-0.349904	-4.781076	-0.673130
H	0.545122	-4.376339	0.812648
H	-0.795373	-5.576089	0.878167

C	-3.206883	-3.902492	-0.063516
H	-4.071827	-3.423671	0.429188
H	-3.296494	-4.989221	0.110165
C	-2.020979	-3.522814	2.580200
H	-1.085407	-3.422330	3.152201
H	-2.736801	-2.754790	2.914652
H	-2.443569	-4.526918	2.750893
O	1.515741	2.785024	3.794949
H	1.274404	2.697766	2.813469
C	2.054286	4.001311	4.006493
H	2.334005	4.113194	5.073604
O	2.216738	4.868538	3.156122
O	1.374223	-3.506093	3.428387
H	1.560667	-2.967410	2.589245
C	2.284939	-4.493484	3.530558
H	2.087952	-5.099965	4.437321
O	3.203518	-4.702509	2.746840

TS7

E=-3389.466501 Hartrees/particle

Mo	-0.040411	-0.191848	-0.140458
Mo	-0.157709	-0.130329	2.673017
Mo	2.450121	-0.128751	1.482764
S	1.551018	1.461276	-0.233471
S	-1.700750	0.819344	1.185294
S	0.840393	-1.910934	1.368052
S	1.435996	1.531447	2.942008
P	-1.247223	1.137339	-1.966612
P	0.973016	-1.266879	-2.365337
P	4.544149	1.300315	1.580688
P	3.433688	-0.798513	3.867273
P	-1.362335	1.163481	4.506546
P	-1.938904	-1.819823	3.445249
H	3.024546	-0.377570	-0.217696
H	5.014073	-3.366556	0.735004
C	4.293532	-2.549215	0.547551
O	4.043897	-1.756907	1.517474
O	3.779915	-2.446230	-0.615439
H	3.196619	-1.281283	-0.399410
C	5.870226	0.850840	0.370708
H	6.163714	-0.200823	0.507126
H	5.486426	0.981540	-0.653236
H	6.752835	1.495194	0.515866
C	4.395251	3.127204	1.362269
H	4.037001	3.340985	0.343125
H	3.663480	3.523291	2.083170
H	5.372229	3.613781	1.518097
C	2.878078	-0.016067	5.444544
H	2.952997	1.079827	5.392402
H	1.829120	-0.288730	5.624391

H	3.510150	-0.392047	6.266300
C	3.458745	-2.584374	4.349440
H	2.426551	-2.901767	4.566482
H	3.860030	-3.189578	3.525934
H	4.077066	-2.723986	5.251676
C	5.236359	-0.292466	3.816594
H	5.771689	-1.027356	3.194538
H	5.648513	-0.349058	4.839315
C	5.379571	1.129493	3.246318
H	4.897812	1.865610	3.914586
H	6.441027	1.418862	3.151825
C	2.555797	-0.596239	-3.068790
H	2.637302	0.477773	-2.838419
H	3.416702	-1.119521	-2.628623
H	2.572851	-0.747257	-4.160967
C	-1.159704	2.980348	-1.918399
H	-1.621695	3.337894	-0.984328
H	-0.107618	3.304069	-1.938178
H	-1.696128	3.411388	-2.779467
C	1.158071	-3.093262	-2.604339
H	1.932532	-3.470952	-1.920092
H	0.205965	-3.601939	-2.390962
H	1.453391	-3.306806	-3.645265
C	-3.057771	0.803868	-2.107090
H	-3.213911	-0.277881	-2.226181
H	-3.557454	1.128697	-1.181006
H	-3.478685	1.350381	-2.966747
C	-0.251984	-0.811711	-3.702925
H	-1.154906	-1.423792	-3.536215
H	0.173103	-1.093524	-4.682485
C	-0.588436	0.686976	-3.652778
H	0.311693	1.301015	-3.833514
H	-1.333068	0.959751	-4.420983
C	-1.969914	2.861034	4.102456
H	-1.109131	3.501480	3.852526
H	-2.638595	2.813141	3.229227
H	-2.509099	3.291894	4.962076
C	-0.453964	1.440366	6.093983
H	-0.208061	0.474003	6.560439
H	0.482663	1.981812	5.891264
H	-1.077980	2.030179	6.785395
C	-3.483404	-2.062586	2.448381
H	-3.956197	-1.086957	2.256321
H	-3.243333	-2.525155	1.479124
H	-4.184540	-2.713944	2.996784
C	-1.388593	-3.550174	3.776764
H	-1.066851	-4.019200	2.834126
H	-0.534752	-3.530834	4.471182
H	-2.212701	-4.136533	4.215081
C	-2.612986	-1.259356	5.092596

H	-1.854804	-1.505762	5.856675
H	-3.523687	-1.837162	5.327815
C	-2.893609	0.250976	5.062877
H	-3.702121	0.488232	4.348909
H	-3.206031	0.626514	6.052822
O	-1.518661	-1.611253	-0.855407
C	-1.671372	-2.887421	-0.579030
H	-0.827912	-3.345767	-0.005169
O	-2.660678	-3.557852	-0.909543
H	0.309199	-1.097183	4.055880

TS8

E=-3388.296226 Hartrees/particle

Mo	0.274904	1.722290	0.108564
Mo	1.607754	-0.781062	0.200204
Mo	-1.173402	-0.742885	-0.169993
S	-1.243585	1.198295	-1.578652
S	2.327392	1.138958	-0.927228
S	-0.015013	-0.007471	1.796602
S	0.387449	-1.899201	-1.467217
P	0.773159	3.930027	-1.117145
P	-1.603939	3.375193	1.034738
P	-2.900503	-1.704859	-1.775003
P	-1.538586	-3.161669	0.862951
P	3.537845	-2.060377	-0.841789
P	3.427747	-0.526544	1.995071
H	-2.581341	-1.141750	1.098864
C	-3.340171	-0.266141	1.547627
O	-3.822680	-0.613169	2.628215
O	-3.409358	0.702699	0.762078
C	-0.830299	5.071346	0.894303
H	0.018894	5.121262	1.594860
H	-1.571996	5.831050	1.197493
C	-0.363887	5.304553	-0.552865
H	-1.225221	5.331487	-1.242500
H	0.165329	6.267665	-0.655639
C	-3.170491	3.634604	0.081936
H	-2.966655	3.635621	-0.999810
H	-3.858005	2.808817	0.304817
H	-3.620567	4.597413	0.376253
C	-2.201810	3.304156	2.783407
H	-2.777984	2.376781	2.921783
H	-1.353379	3.306980	3.483520
H	-2.848172	4.171653	2.997077
C	2.452912	4.619624	-0.779561
H	2.578065	4.736759	0.306822
H	3.219843	3.923357	-1.151813
H	2.560378	5.594923	-1.281520
C	0.656579	3.957774	-2.957923
H	1.382207	3.241330	-3.374738

H	-0.355315	3.656141	-3.269295
H	0.877709	4.966989	-3.341950
C	4.415224	1.036385	2.121319
H	3.783770	1.865406	2.473927
H	5.245498	0.887782	2.832125
H	4.822141	1.299810	1.133155
C	2.939456	-0.865175	3.742094
H	2.226305	-0.100049	4.085529
H	2.450685	-1.850140	3.793770
H	3.827727	-0.854370	4.394505
C	4.746643	-1.801586	1.658014
H	5.632981	-1.572120	2.274415
H	4.352769	-2.778783	1.988092
C	5.091558	-1.820959	0.161775
H	5.812696	-2.620925	-0.079927
H	5.540942	-0.862516	-0.153104
C	4.035364	-1.634204	-2.567915
H	3.195248	-1.844915	-3.248658
H	4.275144	-0.561203	-2.625982
H	4.911720	-2.228378	-2.874548
C	3.346511	-3.897555	-0.902505
H	3.201760	-4.288711	0.116348
H	2.461983	-4.154386	-1.505634
H	4.242324	-4.357863	-1.350649
C	-4.612365	-1.010035	-1.702272
H	-5.050111	-1.154860	-0.703978
H	-4.573668	0.069456	-1.912703
H	-5.242184	-1.512632	-2.454588
C	-2.520136	-1.641418	-3.579900
H	-2.475617	-0.588709	-3.900731
H	-1.543556	-2.111678	-3.770568
H	-3.302969	-2.166091	-4.151535
C	-3.130602	-3.526627	-1.426578
H	-2.305882	-4.053869	-1.938557
H	-4.074743	-3.868454	-1.885181
C	-0.314075	-4.504819	0.531312
H	-0.203997	-4.668578	-0.551062
H	0.662640	-4.207752	0.941606
H	-0.659514	-5.434238	1.013020
C	-3.107537	-3.818328	0.083988
H	-3.963612	-3.343638	0.595416
H	-3.175701	-4.903098	0.277659
C	-1.827988	-3.301033	2.680893
H	-0.887827	-3.070383	3.206573
H	-2.597656	-2.575406	2.989282
H	-2.149219	-4.323526	2.938771
O	1.256377	2.904432	1.588711
C	1.356657	2.768967	2.896935
H	0.681332	1.995805	3.340124
O	2.130901	3.428711	3.600186

H	1.800394	-2.143694	1.281219

TS9

E=-3579.245048 Hartrees/particle

Mo	0.062356	-0.165999	0.005974
Mo	-0.045946	-0.308770	2.817944
Mo	2.554774	-0.139110	1.506190
S	1.658550	1.495116	-0.132645
S	-1.552377	0.766326	1.387118
S	0.949151	-1.980139	1.371029
S	1.493566	1.400753	3.067509
P	-1.265339	1.094554	-1.754686
P	0.885663	-1.310880	-2.196749
P	4.576864	1.411597	1.514274
P	3.733357	-0.741561	3.854568
P	-1.355473	1.055752	4.567212
P	-2.188710	-1.838239	3.296261
H	-1.108838	-1.327998	-0.648489
H	3.213257	-0.278853	-0.276087
H	5.246579	-3.241129	0.848904
C	4.386866	-2.583283	0.588378
O	4.203823	-1.634574	1.465860
O	3.733688	-2.781352	-0.455640
H	3.012761	-1.068289	-0.241099
C	5.818682	1.048792	0.192177
H	6.153973	0.003946	0.277059
H	5.356061	1.195634	-0.796599
H	6.685460	1.722590	0.291035
C	4.311890	3.230430	1.353434
H	3.877703	3.451894	0.366311
H	3.610676	3.568156	2.132311
H	5.271160	3.763098	1.461020
C	3.286230	0.117418	5.434470
H	3.525354	1.189188	5.359138
H	2.214509	0.013824	5.643303
H	3.872098	-0.324340	6.257865
C	3.940121	-2.513850	4.345460
H	2.949386	-2.975062	4.460178
H	4.491371	-3.039809	3.554115
H	4.497764	-2.584079	5.293727
C	5.503264	-0.160881	3.664197
H	6.009856	-0.871630	2.992945
H	5.996845	-0.208101	4.651143
C	5.555433	1.265767	3.097420
H	5.116598	1.988141	3.808534
H	6.594771	1.587319	2.909434
C	2.485438	-0.791985	-2.983457
H	2.592805	0.302741	-2.941033
H	3.326776	-1.259937	-2.451463
H	2.500187	-1.123064	-4.035194

C	-1.207495	2.940841	-1.734053
H	-1.639193	3.306780	-0.789001
H	-0.162338	3.280323	-1.799696
H	-1.783869	3.350073	-2.580033
C	0.988088	-3.151163	-2.278709
H	1.806502	-3.474961	-1.618018
H	0.045706	-3.603488	-1.937561
H	1.196377	-3.469911	-3.313534
C	-3.073136	0.728254	-1.817108
H	-3.220161	-0.345176	-2.017247
H	-3.533649	0.978414	-0.848523
H	-3.548188	1.325685	-2.612725
C	-0.353996	-0.863193	-3.518680
H	-1.263160	-1.465909	-3.343827
H	0.058549	-1.149874	-4.501830
C	-0.673834	0.638398	-3.462705
H	0.224466	1.245403	-3.673584
H	-1.444506	0.916930	-4.202740
C	-1.572730	2.855019	4.203012
H	-0.591028	3.352566	4.242548
H	-1.997984	2.989169	3.196600
H	-2.241907	3.310124	4.951729
C	-0.746635	1.091432	6.310869
H	-0.738362	0.069947	6.716906
H	0.277531	1.497492	6.328605
H	-1.402476	1.736612	6.918669
C	-3.480415	-1.926374	1.972275
H	-3.817681	-0.916991	1.693920
H	-3.075623	-2.424870	1.080688
H	-4.337048	-2.508340	2.351695
C	-1.983695	-3.629869	3.705448
H	-1.477149	-4.130433	2.865018
H	-1.380625	-3.734694	4.616658
H	-2.973162	-4.091749	3.859941
C	-3.162461	-1.107218	4.715990
H	-2.704767	-1.494385	5.638318
H	-4.204343	-1.467092	4.654534
C	-3.109640	0.429700	4.683165
H	-3.639344	0.825463	3.797596
H	-3.590637	0.864588	5.576997
O	-2.203706	-3.556114	-0.826644
H	-1.773093	-2.636917	-0.775674
C	-3.068504	-3.593125	-1.858650
H	-3.556707	-4.587288	-1.908576
O	-3.293521	-2.676420	-2.640031
O	0.798471	-1.550018	4.360047
C	0.526091	-2.059073	5.531036
H	1.436423	-2.487172	6.017203
O	-0.570772	-2.123942	6.112271

TS10

E=-3578.073601 Hartrees/particle

Mo	0.198466	1.571356	0.129293
Mo	1.505580	-0.927735	0.387490
Mo	-1.288378	-0.851569	-0.164551
S	-1.310424	1.084311	-1.579249
S	2.243826	0.940104	-0.811464
S	-0.202675	-0.063001	1.849589
S	0.349726	-1.987105	-1.384571
P	0.866802	3.802018	-0.908149
P	-1.535344	3.244274	1.142411
P	-3.008853	-1.713137	-1.840480
P	-1.770016	-3.292335	0.759172
P	3.550672	-2.048371	-0.672194
P	3.453035	-0.256647	2.049886
H	0.882613	2.537078	1.439286
H	-2.700491	-1.265455	1.076496
C	-3.478611	-0.394908	1.501078
O	-4.005737	-0.757541	2.554179
O	-3.511700	0.584274	0.724084
C	-0.727110	4.924393	1.099415
H	0.112211	4.927589	1.816114
H	-1.456714	5.687821	1.421803
C	-0.230286	5.199038	-0.329272
H	-1.076552	5.280664	-1.033466
H	0.336178	6.144561	-0.387520
C	-3.115431	3.589640	0.242529
H	-2.914941	3.731719	-0.830250
H	-3.787547	2.730814	0.365445
H	-3.579174	4.500680	0.656327
C	-2.087769	3.033983	2.890983
H	-2.687165	2.113852	2.964912
H	-1.217450	2.948111	3.558592
H	-2.703687	3.895778	3.196997
C	2.550179	4.391880	-0.427744
H	2.593469	4.490482	0.668831
H	3.308410	3.662767	-0.751853
H	2.754534	5.367839	-0.898004
C	0.859907	3.936801	-2.749253
H	1.584103	3.218113	-3.164521
H	-0.142364	3.687567	-3.131090
H	1.135318	4.956710	-3.063940
C	4.080198	1.478446	1.898069
H	4.557112	1.617240	0.916200
H	3.253902	2.197906	1.979955
H	4.820568	1.666229	2.693124
C	3.223156	-0.439360	3.871972
H	2.467205	0.288107	4.207164
H	2.890609	-1.461940	4.095073

H	4.176199	-0.237364	4.389083
C	4.979324	-1.241269	1.618521
H	5.860910	-0.738703	2.053794
H	4.855033	-2.224986	2.096382
C	5.117955	-1.381476	0.093717
H	5.957688	-2.047903	-0.170845
H	5.314397	-0.403748	-0.381739
C	3.810032	-1.803286	-2.483625
H	2.997604	-2.299808	-3.037160
H	3.794878	-0.728575	-2.721331
H	4.776525	-2.236843	-2.788821
C	3.698469	-3.880148	-0.481902
H	3.738558	-4.139071	0.586240
H	2.825973	-4.370681	-0.941395
H	4.616574	-4.229638	-0.982492
C	-4.617672	-0.805377	-1.904285
H	-5.130179	-0.854569	-0.932764
H	-4.420676	0.251735	-2.138863
H	-5.258233	-1.249462	-2.683822
C	-2.504130	-1.772880	-3.614610
H	-2.308265	-0.748494	-3.968427
H	-1.582154	-2.365431	-3.716841
H	-3.304950	-2.224063	-4.223122
C	-3.492661	-3.478144	-1.452241
H	-2.785498	-4.132144	-1.992170
H	-4.497597	-3.677522	-1.862821
C	-0.730931	-4.722109	0.216638
H	-0.796532	-4.845010	-0.874878
H	0.317131	-4.540326	0.488104
H	-1.095125	-5.638536	0.709862
C	-3.442084	-3.759461	0.060034
H	-4.211919	-3.181178	0.600574
H	-3.631477	-4.827057	0.268857
C	-1.969916	-3.522294	2.579577
H	-0.998089	-3.343509	3.063928
H	-2.699131	-2.785713	2.954376
H	-2.319240	-4.542889	2.806699
O	1.313801	2.685664	3.914415
H	1.134669	2.587015	2.922650
C	1.917149	3.872377	4.124195
H	2.132809	4.004936	5.203473
O	2.192817	4.693996	3.257819
O	1.334893	-2.644591	1.651637
C	1.952532	-3.504326	2.416097
H	1.289195	-4.359282	2.694877
O	3.119341	-3.458963	2.839034

TS11

E=-3578.067083 Hartrees/particle

Mo	0.023668	-0.336681	0.028770

Mo	-0.066596	-0.338326	2.866098
Mo	2.526363	-0.214572	1.555971
S	1.573285	1.357312	-0.100900
S	-1.510482	0.753814	1.407560
S	0.963016	-2.057918	1.489019
S	1.481521	1.394206	3.038150
P	-1.285136	1.015578	-1.726101
P	0.991697	-1.288517	-2.282991
P	4.544275	1.334175	1.451926
P	3.734228	-0.706336	3.906972
P	-1.344027	1.067893	4.595985
P	-2.251943	-1.798415	3.325320
H	3.031106	-0.493624	-0.172933
H	5.154357	-3.377147	0.788222
C	4.389574	-2.600274	0.600908
O	4.168825	-1.770693	1.551163
O	3.806685	-2.575774	-0.529688
H	3.193785	-1.381989	-0.320405
C	5.760580	0.921694	0.120722
H	6.139473	-0.101856	0.263109
H	5.262485	0.982373	-0.859558
H	6.605480	1.629059	0.147005
C	4.270759	3.144571	1.220405
H	3.825731	3.324972	0.229818
H	3.576834	3.511316	1.992638
H	5.229697	3.683153	1.296199
C	3.286765	0.222063	5.442970
H	3.486602	1.295970	5.309167
H	2.221058	0.088535	5.667694
H	3.897344	-0.158573	6.278799
C	3.944142	-2.451376	4.484931
H	2.953986	-2.898158	4.651039
H	4.469927	-3.023552	3.708267
H	4.526110	-2.474011	5.420828
C	5.501746	-0.138682	3.663100
H	5.999588	-0.883595	3.021908
H	6.011563	-0.139631	4.642764
C	5.550148	1.258238	3.025591
H	5.126098	2.016818	3.707382
H	6.587948	1.564361	2.806122
C	2.557815	-0.573287	-2.975865
H	2.628267	0.496582	-2.725701
H	3.427352	-1.094743	-2.549578
H	2.571756	-0.703472	-4.070698
C	-1.291395	2.854931	-1.591828
H	-1.762208	3.145451	-0.639300
H	-0.257415	3.232554	-1.604834
H	-1.857672	3.295382	-2.428526
C	1.210191	-3.099083	-2.605306
H	1.990934	-3.496081	-1.939282

H	0.269260	-3.639460	-2.425569
H	1.512910	-3.250008	-3.654984
C	-3.076808	0.584973	-1.835127
H	-3.178722	-0.494585	-2.016359
H	-3.567344	0.825315	-0.879111
H	-3.551415	1.157214	-2.648807
C	-0.265589	-0.804239	-3.577176
H	-1.146695	-1.453294	-3.434573
H	0.153780	-1.022527	-4.574939
C	-0.649637	0.676786	-3.445266
H	0.224396	1.330148	-3.615263
H	-1.422705	0.959942	-4.180911
C	-1.532070	2.868899	4.228829
H	-0.542369	3.350408	4.267483
H	-1.952546	3.005260	3.220709
H	-2.195030	3.337202	4.974829
C	-0.721227	1.089623	6.335456
H	-0.729707	0.070226	6.747158
H	0.308852	1.479776	6.350299
H	-1.364046	1.744403	6.946905
C	-3.568478	-1.764141	2.021522
H	-3.968107	-0.742596	1.929670
H	-3.166193	-2.065967	1.044968
H	-4.381635	-2.446523	2.321029
C	-2.108355	-3.605684	3.686198
H	-1.643936	-4.107634	2.822772
H	-1.481014	-3.747081	4.576636
H	-3.108827	-4.034757	3.862273
C	-3.180977	-1.066588	4.772563
H	-2.708067	-1.464637	5.682597
H	-4.229150	-1.411292	4.731478
C	-3.106646	0.469847	4.739580
H	-3.646696	0.875399	3.864659
H	-3.565652	0.911603	5.641572
O	-1.423460	-1.778749	-0.632906
C	-1.446985	-3.094570	-0.529673
H	-0.544742	-3.541334	-0.045810
O	-2.385597	-3.799864	-0.920309
O	0.746881	-1.489641	4.482368
C	0.472464	-2.042285	5.632776
H	1.398054	-2.374275	6.163570
O	-0.639347	-2.231238	6.154411

TS12

E=-3576.896465 Hartrees/particle

Mo	0.257051	1.584524	0.222409
Mo	1.555155	-0.943066	0.360123
Mo	-1.240889	-0.825276	-0.145265
S	-1.206583	1.123974	-1.516425
S	2.248949	0.915315	-0.843755

S	-0.132657	-0.139746	1.880220
S	0.352087	-1.942153	-1.412642
P	0.864258	3.786779	-0.976985
P	-1.585441	3.291227	1.090326
P	-2.992546	-1.610642	-1.833742
P	-1.764112	-3.282200	0.705811
P	3.527342	-2.113006	-0.794283
P	3.606627	-0.326834	1.935646
H	-2.663666	-1.237100	1.081441
C	-3.419548	-0.365094	1.548924
O	-3.944660	-0.758894	2.590951
O	-3.435202	0.641985	0.809400
C	-0.763179	4.964419	0.994584
H	0.060911	4.982898	1.726014
H	-1.493825	5.741447	1.279594
C	-0.239306	5.195836	-0.432319
H	-1.074360	5.264388	-1.151142
H	0.330015	6.138490	-0.504804
C	-3.113355	3.583705	0.088936
H	-2.870981	3.613731	-0.984322
H	-3.813979	2.757942	0.266849
H	-3.567991	4.541176	0.393432
C	-2.236176	3.206362	2.817676
H	-2.832486	2.288112	2.925645
H	-1.411078	3.187850	3.544660
H	-2.873831	4.083006	3.019021
C	2.559903	4.404232	-0.588018
H	2.668415	4.487115	0.503259
H	3.309466	3.693046	-0.967538
H	2.712588	5.388225	-1.060253
C	0.794726	3.832843	-2.819390
H	1.503466	3.095288	-3.228070
H	-0.219805	3.573086	-3.158446
H	1.061778	4.837323	-3.186009
C	4.335508	1.360608	1.712037
H	4.730253	1.463140	0.689594
H	3.578724	2.140297	1.877166
H	5.161448	1.484760	2.432271
C	3.462698	-0.466316	3.770966
H	2.765895	0.303133	4.137333
H	3.089949	-1.467144	4.027881
H	4.451163	-0.306697	4.233409
C	5.050699	-1.408146	1.458408
H	5.973455	-0.971390	1.879196
H	4.872151	-2.387270	1.929225
C	5.145258	-1.531711	-0.070426
H	5.944276	-2.233701	-0.366856
H	5.375125	-0.557455	-0.537635
C	3.734734	-1.860830	-2.610187
H	2.886089	-2.319489	-3.141287

H	3.755728	-0.784104	-2.838089
H	4.672926	-2.329764	-2.949151
C	3.588705	-3.950014	-0.613502
H	3.693994	-4.214776	0.448918
H	2.658539	-4.391148	-1.004718
H	4.447715	-4.347728	-1.178296
C	-4.591836	-0.684474	-1.833563
H	-5.101517	-0.790144	-0.864896
H	-4.387691	0.383796	-2.001967
H	-5.240481	-1.073974	-2.635329
C	-2.510510	-1.602415	-3.614715
H	-2.315370	-0.565799	-3.931136
H	-1.593276	-2.194799	-3.753502
H	-3.322000	-2.025895	-4.228883
C	-3.487384	-3.383649	-1.504914
H	-2.785260	-4.024659	-2.066492
H	-4.493985	-3.560792	-1.921414
C	-0.732893	-4.704954	0.133461
H	-0.780153	-4.793598	-0.962278
H	0.312058	-4.543779	0.430185
H	-1.116386	-5.631296	0.592328
C	-3.439478	-3.714219	-0.002415
H	-4.204825	-3.147959	0.556710
H	-3.636162	-4.786575	0.171308
C	-1.951373	-3.543302	2.522453
H	-0.975046	-3.375960	3.002296
H	-2.675120	-2.812047	2.917343
H	-2.300593	-4.567370	2.733016
O	1.240716	2.706245	1.746253
C	1.268954	2.569282	3.062337
H	0.548030	1.819467	3.470859
O	2.028890	3.203343	3.800380
O	1.419656	-2.695820	1.565457
C	2.006812	-3.556049	2.354352
H	1.339353	-4.419832	2.592416
O	3.151256	-3.501949	2.831443
